# Effects of Phytotoxic Nonenolides, Stagonolide A and Herbarumin I, on Physiological and Biochemical Processes in Leaves and Roots of Sensitive Plants

**DOI:** 10.3390/toxins15040234

**Published:** 2023-03-23

**Authors:** Elena V. Tyutereva, Anna A. Dalinova, Kirill N. Demchenko, Valeriya A. Dmitrieva, Vsevolod R. Dubovik, Yuriy V. Lukinskiy, Galina V. Mitina, Olga V. Voitsekhovskaja, Alexander Berestetskiy

**Affiliations:** 1 Laboratory of Molecular and Ecological Physiology, Komarov Botanical Institute, Russian Academy of Sciences, 197022 Saint-Petersburg, Russia; etutereva@binran.ru (E.V.T.); ovoitse@binran.ru (O.V.V.); 2 Laboratory of Phytotoxicology and Biotechnology, All-Russian Institute of Plant Protection, Pushkin, 196608 Saint-Petersburg, Russia; adalinova@vizr.spb.ru (A.A.D.);; 3 Laboratory of Cellular and Molecular Mechanisms of Plant Development, Komarov Botanical Institute, Russian Academy of Sciences, 197022 Saint-Petersburg, Russia; demchenko@binran.ru; 4 Laboratory of Microbiological Plant Protection, All-Russian Institute of Plant Protection, Pushkin, 196608 Saint-Petersburg, Russia

**Keywords:** *Cirsium arvense*, *Arabidopsis thaliana*, *Stagonospora cirsii*, phytotoxin, macrolide, mechanism of action, photosynthesis, OJIP kinetics, reactive oxygen species, membrane potential, mitosis

## Abstract

Phytotoxic macrolides attract attention as prototypes of new herbicides. However, their mechanisms of action (MOA) on plants have not yet been elucidated. This study addresses the effects of two ten-membered lactones, stagonolide A (STA) and herbarumin I (HBI) produced by the fungus *Stagonospora cirsii*, on *Cirsium arvense*, *Arabidopsis thaliana* and *Allium cepa*. Bioassay of STA and HBI on punctured leaf discs of *C. arvense* and *A. thaliana* was conducted at a concentration of 2 mg/mL to evaluate phenotypic responses, the content of pigments, electrolyte leakage from leaf discs, the level of reactive oxygen species, Hill reaction rate, and the relative rise in chlorophyll *a* fluorescence. The toxin treatments resulted in necrotic and bleached leaf lesions in the dark and in the light, respectively. In the light, HBI treatment caused the drop of carotenoids content in leaves on both plants. The electrolyte leakage caused by HBI was light-dependent, in contrast with that caused by STA. Both compounds induced light-independent peroxide generation in leaf cells but did not affect photosynthesis 6 h after treatment. STA (10 µg/mL) caused strong disorders in root cells of *A. thaliana* leading to the complete dissipation of the mitochondrial membrane potential one hour post treatment, as well as DNA fragmentation and disappearance of acidic vesicles in the division zone after 8 h; the effects of HBI (50 µg/mL) were much milder. Furthermore, STA was found to inhibit mitosis but did not affect the cytoskeleton in cells of root tips of *A. cepa* and *C. arvense*, respectively. Finally, STA was supposed to inhibit the intracellular vesicular traffic from the endoplasmic reticulum to the Golgi apparatus, thus interfering with mitosis. HBI is likely to have another main MOA, probably inhibiting the biosynthesis of carotenoids.

## 1. Introduction

Weed control is essential for realization of the yield potential of agricultural crops. Therefore, the use of chemical herbicides is a necessary element of highly productive farming systems. However, the prohibition of some toxic active components of pesticides, as well as the rapid evolution of weed resistance, makes the search for new means of weed control an urgent task [1]. For example, the expected ban on glyphosate will lead to the loss of about 10% of the grain crop in Europe [2], and the lack of herbicides with new mechanisms of action (MOA) may lead to a decrease in food production by more than 20% in the near future [3]. Natural compounds can be used directly for weed control as biorational herbicides or indirectly by identification of their molecular targets in plants and the following development of synthetic herbicides acting on them [1,4].

**Figure 1:**
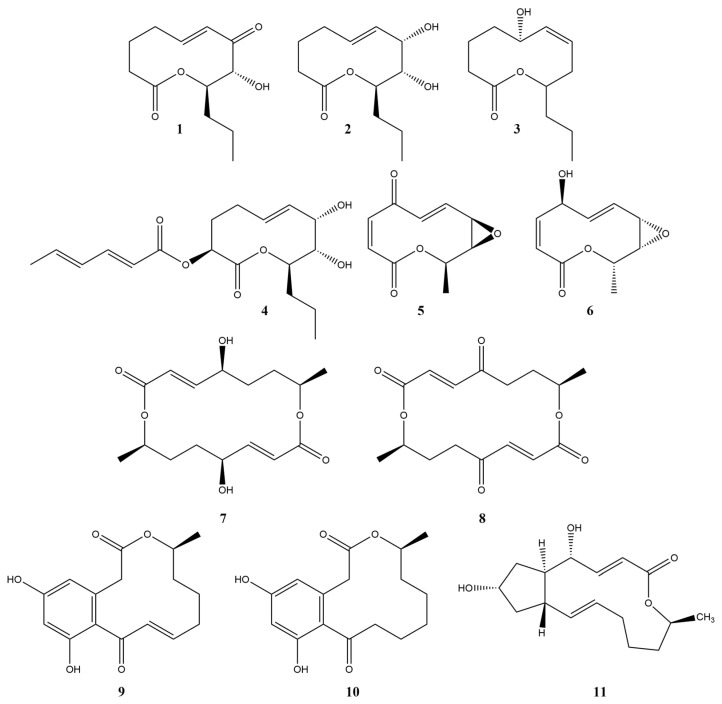
The structure of some phytotoxic macrolides. **1**—stagonolide A, **2**—herbarumin I, **3**—putaminoxin, **4**—pinolidoxin, **5**—pyrenolide A, **6**—stagonolide H, **7**—pyrenophorol, **8**—pyrenophorin, **9**—10,11-dehydrocurvularin, **10**—curvularin, **11**—brefeldin A.

Among phytotoxins of fungal origin, compounds from the group of macrolides (macrolactones) attract attention as prototypes of new herbicides [5]. For example, pyrenophorol (**7**) was proposed as a selective herbicide for the control of wild oat (*Avena sterilis*) [6], with a mechanism of action different from the action of the widely used chemical herbicides as glyphosate, mesotrione, norflurazone, paraquat and diuron [7]. The phytotoxic effects of 10,11-dehydrocurvularin (**9**) resulted in the formation of necrotic leaf lesions, the inhibition of photosynthesis, as well as the inhibition of root growth and disruption of cell division of the root meristem [8,9]. A number of phytotoxic 10-membered lactones are known: pinolidoxin (**4**) [10], putaminoxin (**3**) [11], herbarumin I (**2**) [12,13], stagonolide A (**1**) [14], stagonolide H (**6**) [15], stagonolides J and K [16], and aldaulactone [17]. A complete chemical synthesis of a considerable number of phytotoxic macrolides was attempted, e.g., for pyrenophorol (**7**) [18], 10,11-dehydrocurvularin (**9**) [19], and some nonenolides [20] (Figure 1).

Stagonolide A (STA, **1**) and herbarumin I (HBI, **2**) produced by the phytopathogenic fungus *Stagonospora cirsii* Davis are of particular interest for the development of herbicides and the study of their mechanisms of action. Biotechnological approaches (strain screening, optimization of media composition, fermentation mode and conditions) were used to enhance their production [21,22]. The treatment of aboveground shoots of perennial sowthistle (*Sonchus arvensis*) with solutions of these substances with the adjuvant Hasten^TM^ led to a significant loss of fresh weed biomass and a decrease in the photosynthetic activity of its leaves [23]. Both phytotoxins in micromolar concentrations inhibit the growth of the roots of seedlings of various plants [12,14,16]. In general, STA exhibits higher phytotoxic activity than HBI but at the same time it has a wider spectrum of biological activity [24].

Chemical herbicides and natural phytotoxins are toxic to plants damaging (1) biochemical pathways and physiological processes associated with photosynthesis, (2) critical stages of plant metabolism and (3) their normal growth [25]. Identification of the MOA of a phytotoxic compound is one of the most challenging phases of the development of a novel herbicide. At the initial stage of MOA determination, a panel of simple and informative physiological and biochemical bioassays is used along with the assessment of symptoms, for example, evaluation of photochemical activity, contents of photosynthetic pigments, membrane integrity, mitotic activity, respiratory disorders, uncoupling activity, carbon dioxide uptake, reactive oxygen species (ROS) formation and others [26,27,28]. This approach cannot define a specific molecular target but allows scholars to focus further studies on the range of particular cellular processes. Standard protocols have been developed for the validation of diagnosed MOAs [29]. Metabolomic [27,30], transcriptomic [31] and genomic [32] methods have been adapted for the detection of new MOAs.

In the recent years, new molecular targets for the herbicides, such as solanyl diphosphate synthase, fatty acid thioesterase, plastid peptide deformylase, dihydroxy-acid dehydratase, homogentisate solanesyltransferase and dihydroorotate dehydrogenase, were elucidated [33]. Two mycotoxins, patulin and cytochalasin A, were found to inhibit the photosystem II in *Ageratina adenophora* [34,35], while fumagillin and three other fungal toxins negatively affected several phases of photosynthesis in *Chlamydomonas reinhardtii* [36]. The natural alkaloid berberine was shown to regulate the expression of thalianol and marneral gene clusters in *Arabidopsis thaliana* [37]. Spliceostatin C produced by a bioherbicidal bacterial strain was found to be a spliceosome inhibitor [38].

Despite the considerable number of known phytotoxic nonenolides, their MOAs on plants have not yet been elucidated. A preliminary study of STA, the most potent phytotoxin of the group, showed that it was likely to target plant photosynthesis [39]. Interestingly, STA (**1**) and the structurally similar HBI (**2**) remarkably differed in their toxicity profiles [24]. The aim of this study was the comparison of physiological and biochemical effects of these two substances on sensitive plants. The results provide important information for the further elucidation of molecular targets of STA and HBI.

## 2. Results

### 2.1. Leaf Disorders

#### 2.1.1. Leaf Puncture Assay

**Figure 2:**
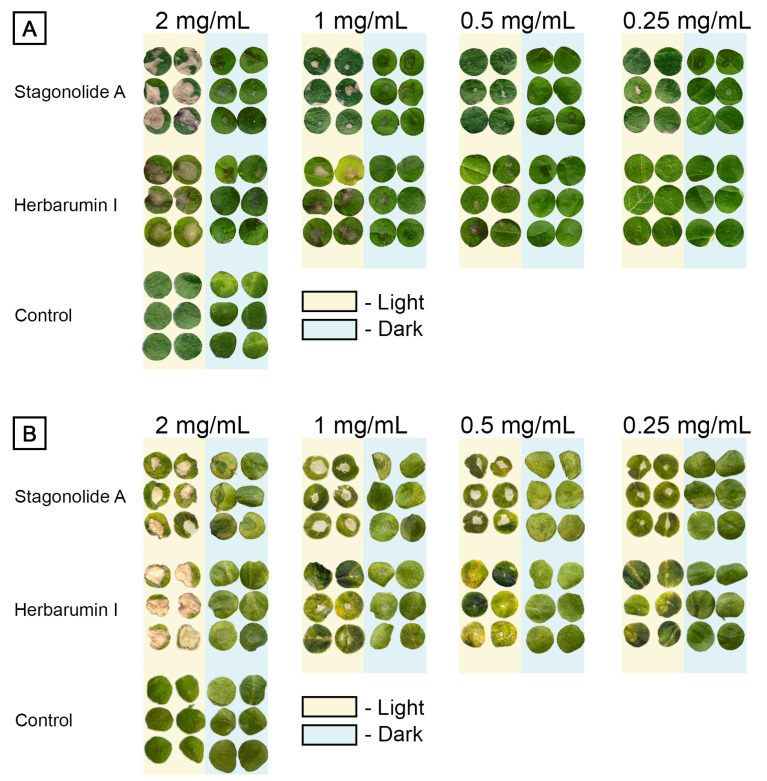
The lesions caused by stagonolide A and herbarumin I on punctured 1-cm leaf discs of *Cirsium arvense* (**A**) and *Arabidopsis thaliana* (**B**) incubated 48 h under continuous light or in the dark.

Both STA and HBI demonstrated phytotoxicity in the leaf puncture assay (Figure 2). Their effects on Canada thistle leaf discs were characterized by necrotic lesions that looked more discolored when treated with STA than with HBI, and more damaged when incubated under the light than in the dark (Figure 2A).

The phytotoxic effects of both nonenolides on Arabidopsis leaves were light-dependent. Toxin-treated Arabidopsis discs that were incubated in the dark displayed few or no observable symptoms. Their exposure to continuous light led to the formation of necrotic lesions (Figure 2B).

**Figure 3:**
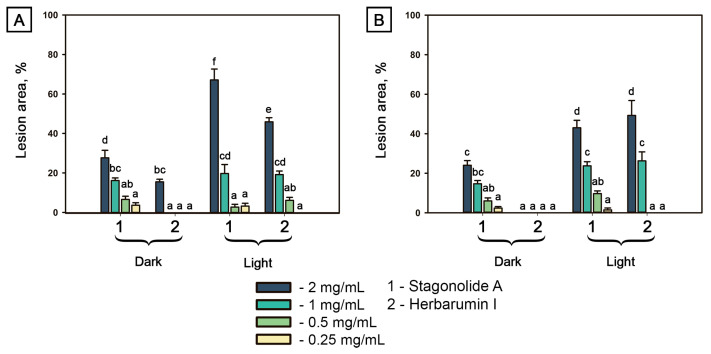
Proportion of necrotic tissue area on punctured 1-cm leaf discs of *Cirsium arvense* (**A**) and *Arabidopsis thaliana* (**B**) after the treatment with stagonolide A and herbarumin I after 48 h incubation under continuous light or in the dark. No effect was observed in the negative control (5% DMSO in water). Different letters indicate significant differences between treatments (*p* < 0.05, Tukey’s post-hoc test).

The relative area of injured leaf surface was used as quantitative indicator of phytotoxicity (Figure 3). Both tested compounds were toxic in a dose-depended manner. In *C. arvense* leaf discs treated with STA at the highest tested concentration of 2 mg/mL, the proportion of the injured leaf surface area of a 1-cm disc (≈0.8 cm^2^) reached 70% if exposed to constant light, but only 30% if incubated in the dark; lower toxin concentrations resulted in similar effects for both light and dark conditions. Phytotoxic effect of HBI (up to 20% of disc area) on *C. arvense* leaf discs incubated in dark was observed only at the highest tested concentration of 2 mg/mL, while in the case of constant light exposure, visible symptoms were observed at concentrations ≤0.5 mg/mL (Figure 3A).

Phytotoxic effects of STA and HBI on Arabidopsis showed similar trends. Exposure of STA-treated leaf discs to constant light led to an increase of toxicity compared with the samples incubated in dark. HBI was not toxic to Arabidopsis without light exposure even at the highest tested concentration, whereas under the light condition, necrotic lesions were formed occupying up to 50% of the disc area at toxin concentration of 2 mg/mL (Figure 3B).

#### 2.1.2. Quantification of Photosynthetic Pigments

**Figure 4:**
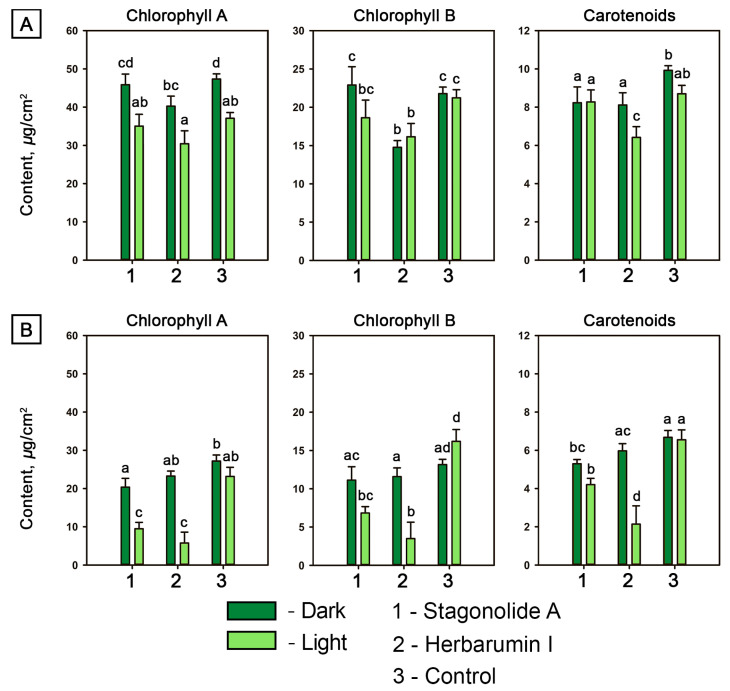
Contents of photosynthetic pigments in toxin-treated (at a concentration of 2 mg/mL) and mock-treated leaf discs of *Cirsium arvense* (**A**) and *Arabidopsis thaliana* (**B**) after 48 h of incubation in the dark or under constant illumination. Different letters indicate significant differences between treatments (*p* < 0.05, Tukey’s post-hoc test).

STA did not cause any significant (ANOVA, *p* < 0.05) decrease in chlorophyll (Chl) contents in *C. arvense* leaf discs neither in the light nor in the dark. Carotenoid contents in STA-treated leaf discs were significantly (by 20%) lower compared to the control under the light condition (Figure 4A). Treatment of *C. arvense* leaf discs with HBI led to a significant reduction of Chl *a* content by 15% in case of dark incubation but not under continuous light. A significant decrease of Chl *b* and carotenoid concentrations was observed in both light and dark conditions in the leaf discs treated with HBI (Figure 4A).

The contents of photosynthetic pigments in Arabidopsis leaf discs depended both on the toxin choice and light conditions (*p* < 0.0001) as well as on the second-order interaction of these two factors (*p* < 0.01). In the dark, STA caused a slight decrease in the level of Chl *a* and carotenoids, while HBI demonstrated no impact on the pigment content in Arabidopsis leaf discs. Under the light condition, the contents of the photosynthetic pigments were lowered to <30% after 48-h incubation of the leaf discs treated with HBI compared to the control treatment. The effects of STA were similar to HBI action, but less dramatic (Figure 4B).

Generally, HBI displayed a stronger (ANOVA, *p* < 0.05) negative effect on the contents of pigments in leaves of both plant species than did STA.

#### 2.1.3. Electrolyte Leakage Assay

**Figure 5:**
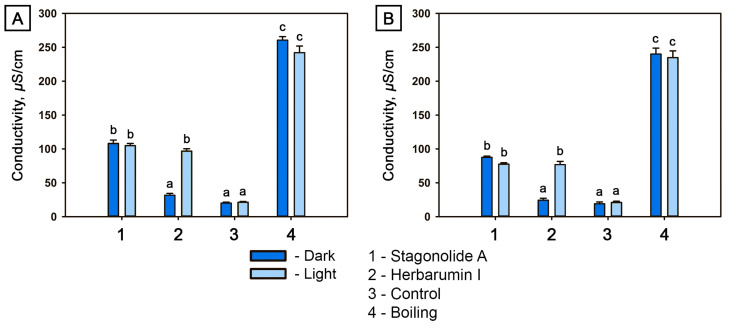
Conductivity of *Cirsium arvense* (**A**) and *Arabidopsis thaliana* (**B**) leaf discs determined 48 h after treatment with stagonolide A or with herbarumin I (at the toxin concentration of 2 mg/mL) in the dark or under constant illumination, respectively. Different letters indicate significant differences between treatments (*p* < 0.05, Tukey’s post-hoc test).

STA induced electrolyte leakage from the leaf discs of both plants under both light and dark conditions. In HBI-treated leaf discs, electrolyte leakage in the dark did not differ from mock-treated leaf discs (at *p* = 0.05) for both plants. However, under light exposure, the HBI-induced electrolyte leakage was comparable to that induced by STA (Figure 5A,B).

#### 2.1.4. Chl *a* Fluorescence Rise Kinetics Assay

**Figure 6:**
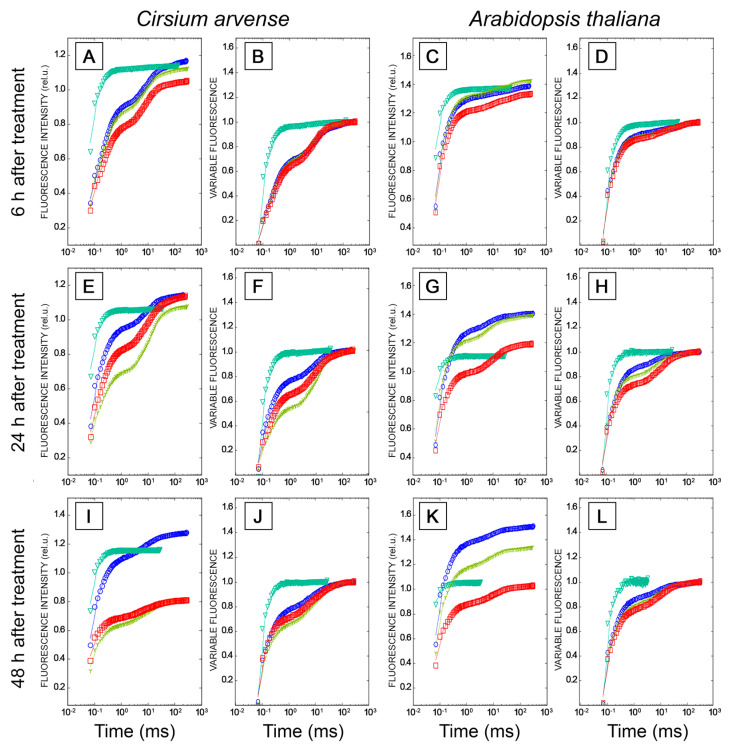
Relative chlorophyll *a* fluorescence rise (OJIP kinetics) in *Cirsium arvense* (**A**,**B**,**E**,**F**,**I**,**J**) and *Arabidopsis thaliana* (**C**,**D**,**G**,**H**,**K**,**L**) leaf discs treated with phytotoxins for 6 h (**A**–**D**), 24 h (**E**–**H**) and 48 h (**I**–**L**). Treatments with mock solution (0.1% DMSO) are marked by blue circles, after leaf treatments with the herbicide diuron (200 µg/mL) by green triangles, treatments with herbarumin I (2 mg/mL) by yellow trefoils, and treatments with stagonolide A (2 mg/mL) by red squares. The fluorescence OJIP curves are plotted on a logarithmic time scale. Time intervals of 6, 24, and 48 h mean the duration of leaf exposure to the light after the application of phytotoxins or mock solution. OJIP curves are plotted as raw fluorescence transients F(t) or as relative variable fluorescence transients V(t). Each curve is the average of three independent experiments, and 10 leaf discs were analyzed in each experiment (*n* = 30).

**Figure 7:**
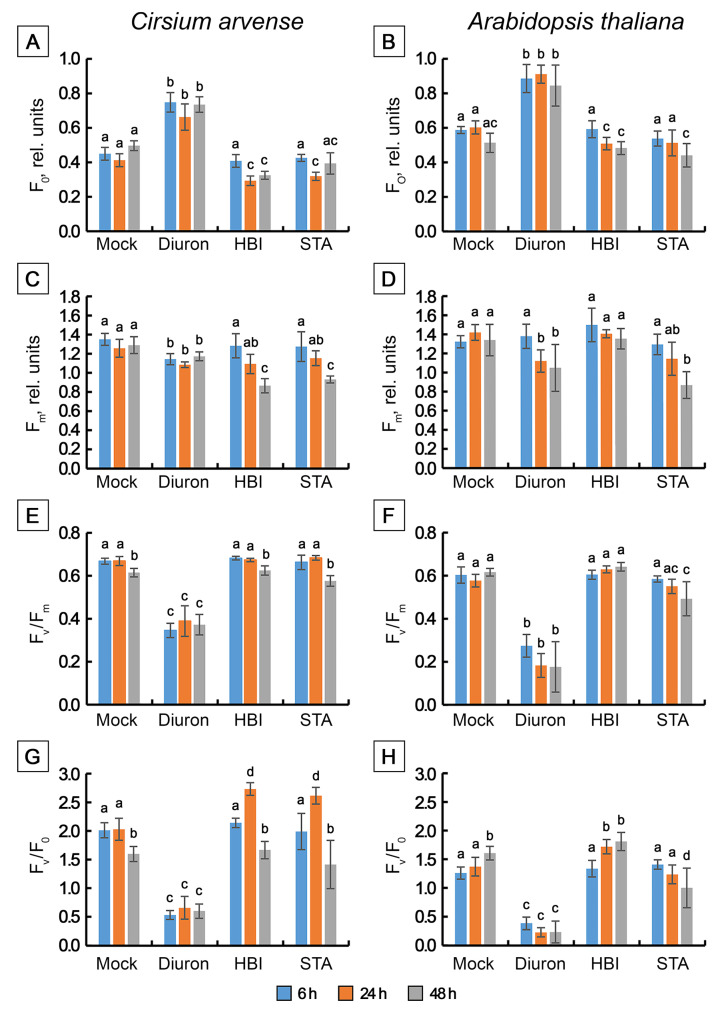
Changes in selected JIP-test fluorescence parameters determined in *Cirsium arvense* and *Arabidopsis thaliana* leaf discs treated with diuron, stagonolide A or herbarumin I. (**A**,**B**) F_O_, (**C**,**D**) F_m_, (**E**,**F**) F_V_/F_m_, (**G**,**H**) Fv/F_0_. Each curve is the average of three independent experiments, and 10 leaf discs were analyzed in each experiment (*n* = 30). Different letters indicate significant differences between treatment groups (*p* < 0.05, Tukey’s post-hoc test).

**Figure 8:**
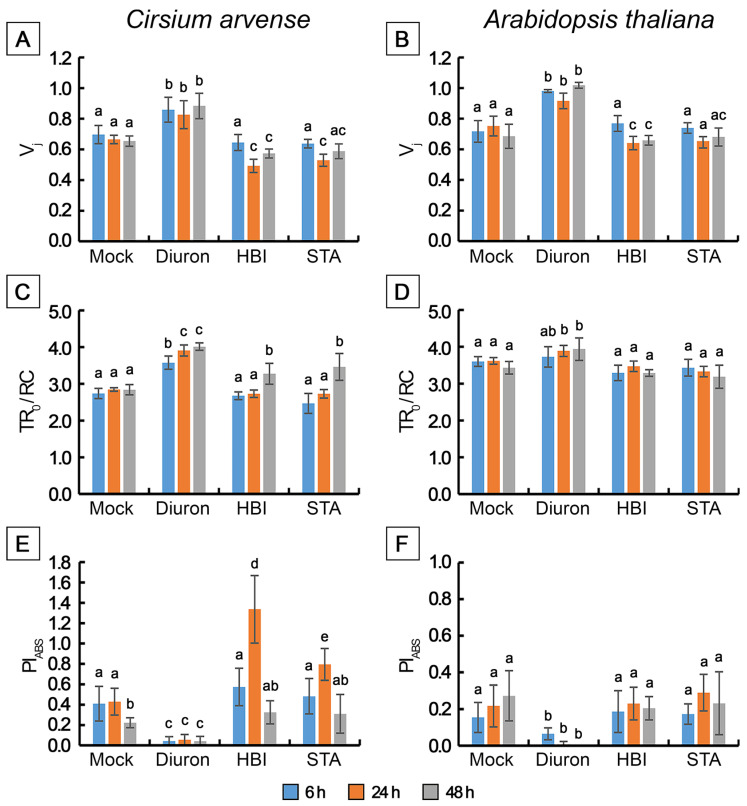
Changes in selected JIP-test fluorescence parameters determined in *Cirsium arvense* and *Arabidopsis thaliana* leaf discs treated with diuron, stagonolide A or herbarumin I. (**A**,**B**) Vj, (**C**,**D**) TR_0_/RC, (**E**,**F**) PI_ABS_. Each point is the average of three independent experiments, and 10 leaf discs were analyzed in each experiment (*n* = 30). Different letters indicate significant differences between treatment groups (*p* < 0.05, Tukey’s post-hoc test).

In *C. arvense* and Arabidopsis leaf discs treated with mock solution, fluorescence induction curves showed polyphasic kinetics with four representative specific points, O-J-I-P, typical for higher plant leaves. The fluorescence intensity increased from F_O_ (minimum or initial fluorescence level, level O, original) up to F_P_ (maximum fluorescence level, step P, peak) through two intermediate local maxima F_J_ (2 ms) and F_I_ (30 ms) [40,41] (Figure 6). Diuron is well-known to cause an increase in the amplitude of the peak J (V_J_) due to the rapid accumulation of reduced Q_A_^−^, and a loss of the IP phase due to the inhibition of electron transfer at the acceptor side of PSII from Q_A_^−^ to Q_B_, and beyond PSII to the photosystem I and further from PSI to ferredoxin-NADP-reductase. When used as a positive control, diuron caused the abovementioned effects already after 6 h after application on punctured leaves of *C. arvense* and Arabidopsis. Apart from these effects, diuron treatment induced an increase in the F_0_ level and a decrease in F_v_/F_m_, F_v_/F_0_ and PI_ABS_ (Figure 6, Figure 7 and Figure 8). The Fm and TR_0_/RC values did not change in Arabidopsis, while in *C. arvense* a decline of Fm and an increase of TR_0_/RC were observed.

Treatment of leaf discs with STA or with HBI for 6 h did not cause significant changes in the shape of OJIP kinetics (Figure 6A–D), or in the values of the main parameters of the JIP test, or in the performance index determined on absorption basis PI_ABS_ compared to the mock-treated leaf discs, in either *C. arvense* or in Arabidopsis (Figure 7 and Figure 8). No changes in the amplitude of V_J_ were observed, which indicates the absence of the effects of HBI and STA on the reduction of the primary electron acceptor Q_A_ (in contrast to diuron). Thus, the leaf treatment with STA and HBI did not disrupt structure or functional activity of PSII during the first 6 h after phytotoxins application.

Exposure of *C. arvense* leaf discs to STA under the light for 24 h led to a decrease in the amplitude of the J peak (Vj), F_0_ and an increase in F_V_/F_0_ and in PI_ABS_ performance index (Figure 6E,F, Figure 7A,G and Figure 8A,E). HBI also caused significant changes in the characteristics of OJIP kinetics, namely, a decrease in F_0_ (Figure 7A) and in the amplitude of the J peak (V_J_) (Figure 6E,F, Figure 7A and Figure 8A). No changes were observed in the maximum fluorescence quantum yield F_V_/F_m_, the indicator of plant photosynthesis performance, caused by both phytotoxins compared to mock treatment (Figure 7E). At the same time, HBI, similar to STA, led to a significant increase in the F_V_/F_0_ parameter (Figure 7G) that reflects the efficiency of the use of excitation energy in PSII and an increase in PI_ABS_ performance index (Figure 8E) that reflects the functional activity of PSII related to the amount of absorbed energy. In Arabidopsis leaves, STA and HBI caused substantial changes in a few OJIP parameters 24 h post treatment. In particular, STA led to a significant decrease in the V_J_ value (Figure 6G,H and Figure 8B). HBI decreased F_0_ and V_J_ values but significantly increased F_V_/F_0_ (Figure 7B,H and Figure 8A).

In *C. arvense* leaf discs, STA treatment for 48 h resulted in a dramatic decrease in F_m_, F_v_/F_m_, F_v_/F_0_ and PI_ABS_ performance index, and in the rise of TRo/RC (Figure 7C,E,G and Figure 8C,E). Compared to the mock controls, HBI caused the changes in numerous parameters of fast chlorophyll *a* kinetics, namely, a drop in F_0_, F_m,_ F_v_/F_m_, F_V_/F_0,_ Vj, PI_ABS_ and an increase in TR_0_/RC (Figure 7A,C,E,G and Figure 8A,C,E). In Arabidopsis, HBI effects after 24 or 48 h of light exposure were similar while STA application resulted in a decrease in F_0_, F_m_, F_v_/F_m_ and F_v_/F_0_ which were not observed in the case of shorter exposition time (Figure 7 and Figure 8).

#### 2.1.5. Hill Reaction

The electron transfer from water to non-physiological oxidants, or the Hill reaction, is a sensitive process and is widely used as an indicator of damage to water-splitting complex. Usually, chloroplasts of spinach (*Spinacea oleracea*) are used in these studies. The activity of the Hill reaction in isolated spinach chloroplasts was not affected by the presence of STA or HBI in reaction mixture. A statistically significant drop of Hill reaction activity was induced only by addition of herbicide diuron (Figure S1).

#### 2.1.6. ROS Assay

To assess the effect of toxins on ROS production, specific fluorescent labels were used: CM-H_2_DCFDA for the analysis of peroxide production, SOSG for the analysis of singlet oxygen production and DHE for the analysis of superoxide production, respectively. Paraquat was used as a positive control for production of superoxide and peroxides, while tenuazonic acid and metribuzin were used as the positive control for singlet oxygen production. In all cases, the experiment was carried out on leaves of *C. arvense* and *A. thaliana*; measurements were carried out 6 and 24 h after treatment with toxins, and effects in the dark and in the light were compared. Changes in the relative intensity of the fluorescence signal of the respective dye (%) are presented in Table 1. The representative photographs and quantitative evaluation of the data are presented in Figures S2–S4 and Table S2 in the Supplementary section.

*Superoxide.* In *C. arvense* leaf discs incubated in toxins for 6 h in the dark, superoxide production was similar to the mock controls. Exposure of toxin-treated leaf discs in the light for 6 h led to an increase of superoxide production only in paraquat-treated discs, consistent with the known MOA for this herbicide: in the light, paraquat rapidly accepts electrons from PSI leading to a strong increase of superoxide levels which are further detoxified to hydrogen peroxide in glutathione-ascorbate cycle. After 24 h of treatment, superoxide production significantly increased regardless of lighting in STA, HBI and paraquat-treated leaf discs (Table 1). In Arabidopsis, treatment of leaf discs with paraquat led to a significant stimulation of superoxide formation at all exposure times and lighting conditions while STA and HBI induced superoxide production only in the dark at both incubation times (Table 1). Pairwise comparison showed that the production of superoxide after treatment with STA and HBI in the light increases after 24 h compared to 6 h exposure; there were no differences in the dark.

*Peroxides.* In *C. arvense* leaf discs incubated in toxins for 6 h in the dark, STA caused a sharp and significant increase in the content of peroxides while HBI and paraquat had no effects. Similar incubation performed in the light led to an increase of peroxide contents in discs treated with STA and HBI but, again, not with paraquat. As peroxides are secondary products of paraquat application following formation of superoxide, and superoxide levels were clearly enhanced upon paraquat application, these data suggest a rapid scavenging of peroxides by paraquat-treated chloroplasts of *C. arvense* during 6 h of treatment. Contrarily, when applied for 24 h, paraquat caused a significant increase in the levels of peroxide production both in the light and in darkness. Both phytotoxins caused a significant increase in the concentrations of peroxide, compared with the mock-treated leaf discs, in the light but not in darkness (Table 1). In a pairwise comparison of the effects of toxins in the light and in the dark, no differences were revealed either after 6 h or after 24 h.

In Arabidopsis leaf discs incubated in toxins for 6 h in the light, paraquat induced a significant and sharp increase in peroxide formation while other toxins did not show any effects. When similar incubation was performed in darkness, only STA caused a significant increase in peroxide as compared to the control. After a 24 h incubation in the dark, a significant increase in peroxide formation was observed in all treatments; similar incubation performed in the light resulted in strong accumulation of peroxides in leaf discs treated with paraquat, and in a moderate increase in leaf discs treated with HBI (Table 1). Similar to the situation found in *C. arvense*, pairwise comparison of the effects of STA in the light and in the dark did not reveal any differences at either exposure time. However, the levels of peroxides in HBI-treated leaf discs significantly increased with time independently of lighting.

*Singlet oxygen.* After 6 h treatment, a significant increase in the production of singlet oxygen was noted in *C. arvense* leaves treated with tenuazonic acid, with the exception of dark incubation during the day. However, the effect of nonenolides and metribuzin on this parameter was insignificant regardless of the terms and conditions of incubation (Table 1). Contrarily, in the leaves of *A. thaliana*, it was found that after 6 h of incubation in the dark, the production of singlet oxygen increased significantly compared to the control when exposed to all the toxicants. In the dark, a significant rise in the level of singlet oxygen was observed for tenuazonic acid and metribuzin. After 24 h post-treatment, no effects on singlet oxygen were detected, regardless of light conditions (Table 1).

### 2.2. Root Disorders

#### 2.2.1. Dynamics of Acidic Compartments as Assessed with Acridine Orange Stain

**Figure 9:**
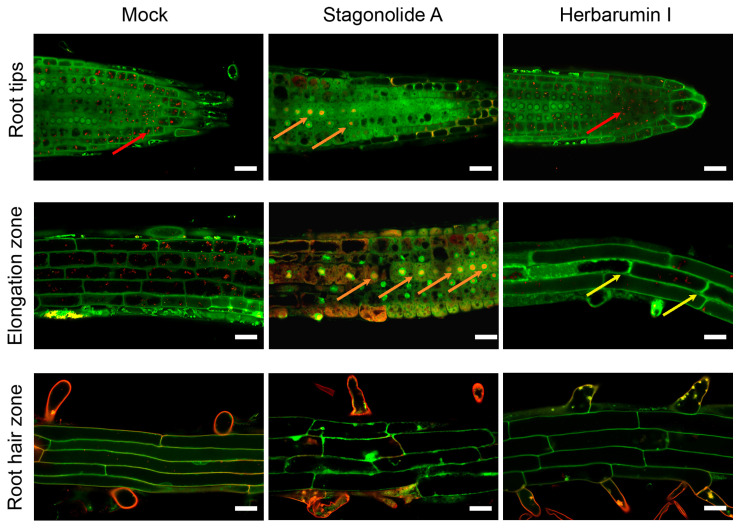
Morphological changes in root cells of Arabidopsis seedlings treated with stagonolide A or herbarumin I. Images were obtained after 8 h of seedlings incubation in phytotoxins solutions or in mock control followed with in vivo staining with acridine orange. Note the dramatic shortening of the length of the cells of roots incubated with stagonolide A (elongation zone) as compared to mock or herbarumin I-treated roots. Two-color merged images were performed by confocal laser scanning microscopy of single optical longitudinal section of Arabidopsis roots. Red channel: λex: 488 nm, λem = 615–660 nm; green channel: λex: 488 nm, λem = 530–540 nm. Red arrows point to the acidic vesicles; orange arrows point to the nucleus with the DNA fragmented in short single-stranded DNA; yellow arrows indicate a partial plasmolysis. Scale bars: 20 µm.

The typical images of root cells of Arabidopsis seedlings after the 8 h incubation with STA (10 µg/mL) or HBI (50 µg/mL) and Acridine Orange (AO) staining are shown in Figure 9.

The cationic dye AO is widely used for the detection of plant cell nuclei, since upon binding to double-stranded DNA it emits fluorescence with a maximum of 525–530 nm (green area), and upon binding to the phosphate group of single-stranded nucleic acids—single-stranded DNA or RNA—the emission maximum shifts to 650 nm (in red wavelength region). AO is effectively used as a vital dye to detect early and late apoptotic nuclei in animal cells [42,43]. In neutral and alkaline solutions, AO has a fluorescence emission maximum at 530 nm, while in acidic conditions the protonated form of the dye emits fluorescence in the orange wavelength range (with an emission maximum at 655 nm) when excited with blue light [44,45]. Inside the cell AO accumulates in acidic cellular compartments, including autophagosomes, and allows to visualize them by epifluorescent and confocal microscopy.

To validate the AO-based semi-quantitative method for assessing the pH, we performed a test for the apoplastic pH of the root cells which can be alkalinized by inhibition of plasma membrane H^+^-ATPase. The specific inhibitor, sodium orthovanadate (Na_3_VO_4_, Sigma-Aldrich), was used as a positive control. Untreated control root hair cells after the staining with AO showed the fluorescence emission in the red region (615–660 nm) which means the acidic pH of apoplast. Treatment with the sodium orthovanadate (100 mM sorbitol, dH_2_O) led to the alkalization of the apoplast and the increased AO fluorescence emission in the green region (530–540 nm). Thus, it was confirmed that the dye can be used for the relative pH assessment of the apoplast of Arabidopsis root cells (Figure S5).

Of two applied phytotoxins, STA had a clear destructive effect on cell components as compared to HBI (Figure 9). Cells of the division and the elongation zones were the most susceptible to STA treatment. The following features were observed: (1) multiple nuclei emitted strong red fluorescence that is indicative of the DNA fragmentation in short single-stranded DNA (ssDNA) fragments in the course of activation of programmed cell death (PCD); (2) disappearance of acidic vesicles (stained bright-red in mock-treated cells) in the division zone; (3) strong inhibition of cell elongation in the elongation zone; (4) a partial separation of the protoplast from the cell walls (partial plasmolysis) in some cells. Incubation of seedlings in the presence of HBI has not resulted in any pronounced decrease in the numbers of acidic vesicles in the cells of root tips, and only led to the partial plasmolysis in few cells of elongation zone (Figure 9).

#### 2.2.2. Mitochondrial Membrane Potential Assay

Mitochondria represent one of the targets of herbicides, being ROS producers and emerging elicitors of PCD not only in animal but also in plant cells. Correct assessment of the relative mitochondrial polarization with the fluorescent membrane potential-sensitive dyes requires at least two fluorescent dyes to be used, one specific to the mitochondrial membrane potential (Δ*Ψ*m) and another one specific to the plasma membrane potential (Δ*Ψ*p) [46]. The latter is necessary to ensure that changes in Δ*Ψ*p are not responsible for poor loading of the mitochondrial dye which would interfere with the analysis.

**Figure 10:**
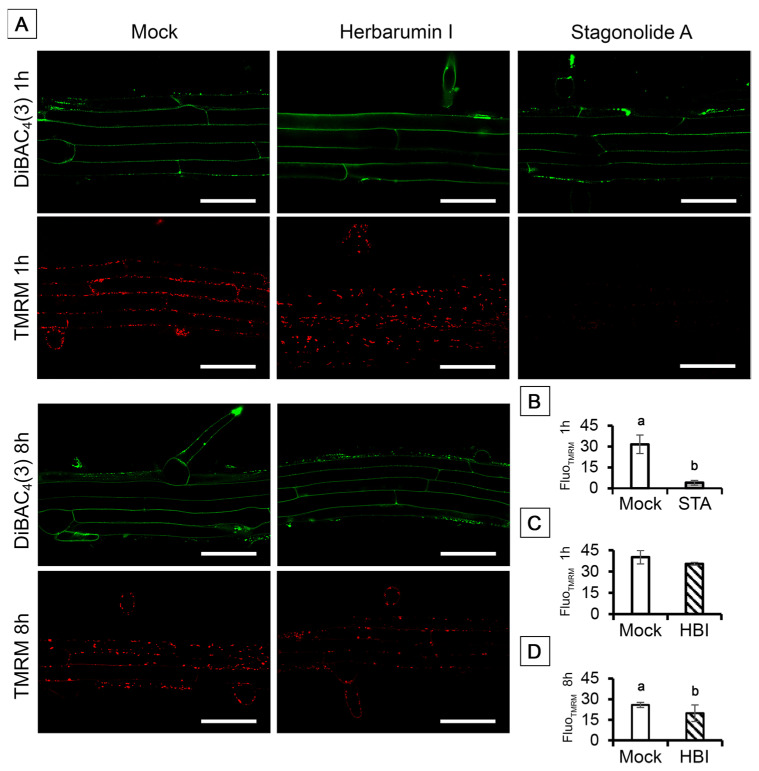
Evaluation of the plasma membrane potential and mitochondrial membrane potential with two membrane potential-sensitive dyes TMRM and DiBAC_4_(3) in Arabidopsis root cells treated with stagonolide A (10 µg/mL) or herbarumin I (50 µg/mL). The lipophilic cationic dye TMRM served as a probe of the mitochondrial potential Δ*Ψ*m, and the lipophilic anionic dye DiBAC_4_(3) was used as a potential sensor of the plasma membrane Δ*Ψ*_P_. (**A**) Confocal images of TMRM and DiBAC_4_(3) stained root cells; (**B**–**D**) Mean intensity of TMRM fluorescence per µm^2^ mitochondria of control and experimental root cells after 1 h of STA or HBI (**B**,**C**) and after 8 h of HBI treatment (**D**). Images were performed by confocal laser scanning microscopy of single optical longitudinal section of Arabidopsis roots. Red channel: λex: 488 nm, λem = 535–605 nm; green channel: λex: 488 nm, λem = 500–545 nm; λex: 488 nm. Different lowercase letters (a,b) indicate statistical significant differences according to Student’s *t*-test (*p* < 0.05). Scale bars: 50 µm.

We used DiBAC_4_(3) to monitor the plasma membrane potential of root cells. In mock-treated roots, DiBAC_4_(3) entered the cells and stick to plasma membrane, visible like a narrow fluorescent band along the inside surface of the cell wall (Figure 10A). It was shown that the pattern of DiBAC4(3) distribution was not disturbed by neither STA nor HBI (Figure 10A). Further, we used the fluorescent lipophilic cationic dye TMRM that accumulates within mitochondria in inverse proportion to the mitochondrial membrane potential (Δ*Ψ*m) according to the Nernst equation. Thus, more polarized mitochondria accumulate more cationic dye, while depolarized mitochondria accumulate less. After only 1 h incubation of Arabidopsis seedlings in the solution of 10 µg/mL STA, mean TMRM fluorescence intensity per unit of mitochondria area dramatically decreased indicating a complete collapse of Δ*Ψ*m (Figure 10A,B). In contrast to this, HBI in a concentration of 50 µg/mL did not significantly affect the mitochondrial membrane potential of root cells after 1 h incubation (Figure 10A,D).

#### 2.2.3. Mitotic Index

The possible effects of STA on the cell cycle and cell divisions were analyzed using onion roots as a model. The mitotic index (MI) of untreated onion roots was at the level of 11%. In the root samples treated with STA, MI was significantly lower: about 2 times at a concentration of 0.1–1 µg/mL STA. Strikingly, this was accompanied by a significant increase in the proportion of cells in anaphase as compared to untreated control where the largest number of dividing cells were in prophase, indicating that STA specifically interferes with anaphase to telophase transition (Table 2).

**Figure 11:**
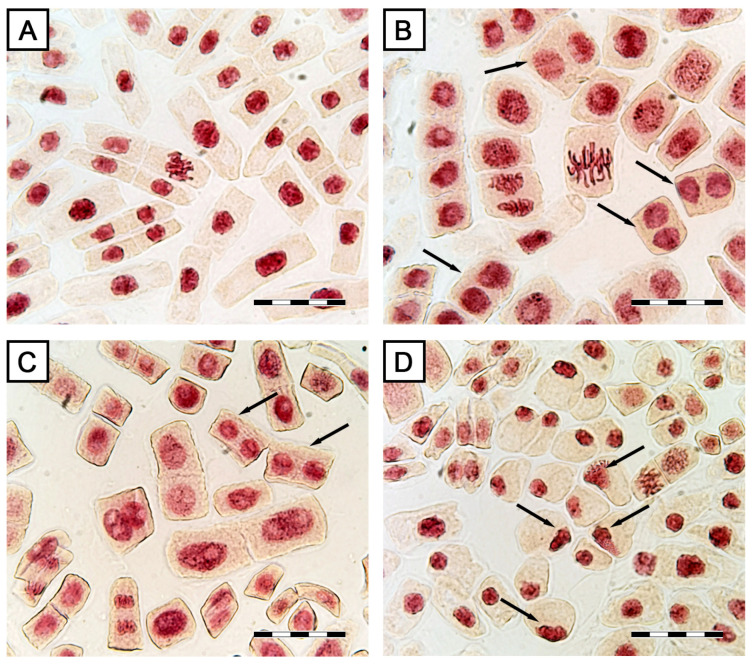
Morphology of onion root tip cells exposed to stagonolide A: (**A**) control—normally dividing cells; (**B**,**C**) binuclear cells (indicated by arrows); (**D**) nuclear fragmentation (indicated by arrows). Scale bar: 50 µm.

The microscopy observations revealed several disorders of cell division caused by the STA action, especially at the telophase stage. The observed binucleation can be a result of failed cytokinesis in dividing cells in all STA treatments (Figure 11B,C). At the maximum STA concentration, cells with partial and complete fragmentation of nuclei were detected (Figure 11D). At the minimal concentration of STA, as well as in the control, cells with light inclusions in nuclei were observed. In the control (treatment with 2% ethanol), no mitotic disorders were detected (Table 2, Figure 11A).

#### 2.2.4. Tubulin and Actin Patterns

Strong effects of STA on mitosis, especially the observed increase of the percentage of cells in the anaphase, prompted us to analyze possible effects of STA on cytoskeleton. To this aim, immunolabeling of tubulin and actin followed by fluorescent microscopy was applied (Figure S6). Notably, STA did not cause any disruption of actin or tubulin cytoskeleton in root tips of *C. arvense* seedlings.

## 3. Discussion

### 3.1. Leaf Disorders

Phenotypic responses of plants to herbicides and natural phytotoxins sometimes result from their secondary effects, rather than the primary mechanism of action (MOA) [26]. Nevertheless, phytotoxic symptoms may shed some light on the MOA. For instance, leaf chlorosis and deformation are caused by inhibitors of pigment biosynthesis and by auxin-type herbicides, respectively, while wilt can be a result of glutamine synthase inhibitors [47,48,49]. Both STA (**1**) and HBI (**2**) caused brown and slightly bleached lesions on punctured *C. arvense* leaf discs in the dark and under the light conditions, respectively (Figure 2A). Phytotoxicity of HBI on *A. thaliana* under light exposure was definitely stronger than in the darkness (Figure 2B and Figure 3B). Leaf lesions is a common effect of many fungal phytotoxins without any specific indication of the MOA. However, the light-dependent leaf damage may be caused by the disruption of photosynthetic electron transport followed by ROS accumulation like in the case of tenuazonic acid affecting PSII [50]. Bleaching lesions may be characteristic for herbicides that inhibit phytoene desaturase [29].

In the light, a decrease of chlorophyll contents in *C. arvense* and Arabidopsis leaves was caused by both STA and HBI that is consistent with formation of lesions. In the dark, STA caused a considerable reduction of Chl *a* in leaves of Arabidopsis but not *C. arvense* while HBI had almost no effect (Figure 4). Despite severe damage of leaves by STA, less than 20% carotene loss was found in light as well as dark conditions in both *C. arvense* and Arabidopsis; at the same time, HBI caused a pronounced effect on the carotenoid contents of leaves of both plants in the light (Figure 4). These data suggest that possible differences between MOA of STA and of HBI are influenced by both plant species and toxin structure. Further quantification of isoprenoids and porphyrins in Arabidopsis leaves will allow to conclude whether the effects of these nonenolides are related with pigment biosynthesis [51,52].

Electrolyte leakage assay is an indirect method to assess plant membrane integrity. Many cell death pathways entail different forms of plasma membrane damage including ROS generation as a result of the release of pore-forming proteins and lysosomal contents, and of other processes which under normal conditions are regulated but can be affected by the toxins [53,54,55]. For instance, paraquat or dinoterb lead to production of ROS that in turn result in light-dependent (paraquat) or light-independent (dinoterb) lipid peroxidation; furthermore, inhibitors of enzymes of lipid biosynthesis such as VLCFA synthase or enoyl-ACP-reductase lead to an increase in membrane permeability [54,56]. In our experiments, STA and HBI demonstrated striking differences: electrolyte leakage induced by STA did not depend on light conditions while that induced by HBI was moderate in the dark but increased dramatically in the light (Figure 5). Although electrolyte leakage observed as long-term effect cannot be interpreted as a result of a direct effect on membrane permeability, but may instead be the result of exposure to secondary damaging agents including intracellular stressors [55,57], these data further support the hypothesis of different action modes of the structurally similar nonenolides.

The abovementioned differences prompted us to analyze photosynthetic performance and ROS production in leaves in response to treatment with STA and HBI. ROS are constantly produced by plant cells, but normally their levels are strictly controlled. It is well known that exposure to many chemical herbicides leads to a significant increase in ROS production; in fact, most herbicides cause ROS production, either as part of the primary or secondary response [56,57,58]. One of the best-studied chemical herbicides in this context is paraquat. It causes severe symptoms of oxidative stress, and the mechanism of its phytotoxic action is well studied: it has been shown that it catalyzes the transfer of electrons from PSI to oxygen, leading to the formation of free radicals [57,58] and contributing to the formation of superoxide [59]. Also, tenuazonic acid has been studied in detail and shown to affect PS II, which eventually leads to an increase in the production of singlet oxygen [60,61]. Our data showed that STA and HBI cause a remarkable increase of ROS production. However, their ROS generation patterns differed from those of paraquat, tenuazonic acid, and metribuzin (Table 1). Notably, no clear induction of ROS was observed in the light. Altogether, our data suggest that the MOAs of these phytotoxins include oxidative stress but are not connected to the disruption of electron flow in PSI and/or PSII. Since both STA and HBI treatment leads to the overproduction of superoxide and peroxides in the dark within, it can be concluded that their effects are not light-dependent, and the production of ROS caused by these nonenolides mainly occurs not in chloroplasts but in other cellular organelles, probably in mitochondria which are the principal source of ROS in darkness and in non-green tissues [62].

We also performed a detailed analysis of the action of STA and HBI on photosynthetic performance using OJIP kinetics and Hill reaction as two most sensitive processes indicative of damage to PSII which is the first indicator of photosynthetic disturbances. Of these two approaches, OJIP kinetics are especially suitable for detection of the very early effects of toxins on photosynthetic performance. The data clearly demonstrated that both donor side and acceptor side of PSII remained unaffected by the action of either STA or HBI (Figure 6, Figure 7 and Figure 8). Importantly, severely inhibition of PSII reaction centers occurred in leaves already after just a few hours of exposure to cytochalasin A (3 h of exposition) [35], tenuazonic acid (6 h of exposition) [63] or diuron (6 h of exposition, Figure 6A–D). The first effects of STA and HBI became visible after 24 h of exposure, thus excluding photosynthesis as primary target of both nonenolides.

### 3.2. Root Disorders

A number of structurally diverse phytotoxic compounds are known to inhibit root growth of sensitive plants and interfere with mitosis [26]. Therefore, the study of the effects of STA (**1**) and HBI (**2**) on root cells might be informative.

Our in vivo analyses of the dynamics of acidic compartments using AO staining and CLSM clearly demonstrated the disappearance of acidic vesicles from the division zone and the elongation zone of Arabidopsis roots subjected to STA (Figure 9). This effect was accompanied by dramatic shortening of the cells from the elongation zone, indicating that the transport of cell wall materials from the Golgi to the apoplast had ceased. These data strongly suggest that STA targets the site of protein transfer from endoplasmic reticulum (ER) to the Golgi, resembling the well-known effect of brefeldin A (BFA, **11**) [64,65].

In animal cells, aberrant accumulation of proteins in ER cisternae leads to release of calcium into the cytoplasm, transport of calcium ions into mitochondria, and induction of PCD [66]. Additionally, in plants, release of calcium from the ER can lead to the accumulation of calcium in mitochondria [67,68]. If STA, similar to BFA, inhibits protein transfer from the ER to Golgi, then this would lead to accumulation of proteins in the ER lumen and development of ER stress which is one of the mechanisms leading to PCD. We did not analyze PCD in our experiments; however, we detected signs of DNA fragmentation in single-stranded fragments in cells of the root elongation zone subjected to STA (Figure 9). Notably, none of the abovementioned effects were observed in root cells exposed to HBI.

Furthermore, STA but not HBI caused complete dissipation of the mitochondrial membrane potential in the root cells as early as after 1 h of incubation of roots (Figure 10), which was the fastest, and one of the strongest, effects revealed by this study. In animal cells, loss of the mitochondrial oxidative phosphorylation due to dissipation of mitochondrial membrane potential Δ*Ψ*m represents a stage of apoptosis which is followed by formation of the mitochondrial permeability transition pore, release of cytochrome c, and proteolytic degradation of the cells [66]. Studies with several model plants showed that PCD-inducing factors can lead to disruption of the mitochondrial electron transport chain and of mitochondrial membrane permeability, followed by a drop in Δ*Ψ*m, release of cytochrome c, generation of ROS and development of oxidative stress [69,70]. Induction of peroxide formation by STA in darkness suggests a similar scenario.

At last, we analyzed cell divisions in onion root cells subjected to STA. A dramatic decrease in the number of mitotic cells, especially those found in the telophase, was detected, while the number of cells undergoing anaphase was highly increased. This suggested that the anaphase-to-telophase transition is affected, and one of the candidate mechanisms might be the inhibition of the cytoskeleton which is necessary for the transfer of anaphase chromosomes to the cell poles. However, immunofluorescence analysis did not reveal any effects of STA on actin or tubulin (Figure S5).

The cessation of formation of Golgi transport vesicles can convincingly explain the observed arrest of mitotic cells in the anaphase: it was shown that BFA-sensitive intracellular vesicular transport is required for spindle positioning before anaphase II in mouse oocytes [71], while in plant cells, the formation of the middle lamella during the telophase relies on vesicular traffic from the Golgi and thus is probably inhibited by STA, preventing the beginning of the telophase and further cytokinesis.

### 3.3. Mechanism of Action Hypotheses

Currently, the mechanisms of action (MOAs) have been elucidated only for a limited number of fungal phytotoxins, and hardly anything is known about the molecular targets of nonenolides in various organisms, including plants. The information on the spectrum of biological activity of phytotoxic macrolides (Figure 1) could give first hints to their hypothetical MOA.

STA (**1**), one of the best characterized nonenolides, has a wide range of biological activity, including phytotoxic, cytotoxic and entomotoxic, as well as a weak antimicrobial activity. HBI (**2**) proved to be selectively phytotoxic with a weak entomotoxicity and with a lack of cytotoxic and antimicrobial activity [14,16,24]. Hence, molecular targets STA may well be biomolecules and processes that are present not only in plants, but also in non-photosynthetic organisms (such as animals), while the target of HBI might be plant-specific.

The well-known phytotoxic macrolides, such as 10,11-dehydrocurvularin (DHC, **9**), pyrenophorol (PNL, **7**) and BFA (**11**), also display a wide range of biological activity, including cytotoxic and antimicrobial properties [72,73,74,75]. Interestingly, as in the case of the structurally similar macrolides STA (**1**) and HBI (**2**), the toxicological profiles of pyrenophorin (PNN, **8**) and PNL (**7**) [76], DHC (**9**) and curvularin (**10**) differ significantly [9,77]. Notably, in all cases mentioned the compound containing a ketone that is conjugated with an adjacent double bond (STA, PNN and DHC) is more toxic than its congener.

The molecular targets of PNL (**7**) and PNN (**8**) in pro- and eukaryotic cells have not been determined yet. The MOA of DHC (**9**) is known only in relation to animal cells: it is STAT3, a signaling protein and activator of transcription [78] as well as an ATP–citrate lyase responsible for lipid biosynthesis [79]. The molecular target for BFA is the same in all eukaryotic cells, representing the Sec 7-type GEF protein, which is necessary for the activation of Arf1 GTPase. Its stabilization on Golgi membranes caused by BFA disrupts the protein transport between the endoplasmic reticulum and the Golgi apparatus [64,65].

It is tempting to speculate that the MOA of STA (**1**), a nonenolide with a strong phytotoxic effect, relies on the inhibition of the intracellular vesicular traffic from the ER to Golgi, which results in ER stress leading to PCD. The dramatic loss of the mitochondrial membrane potential observed already after 1 h of exposure to STA should represent the very first detected sign of the rapidly developing ER stress due to the inhibition of ER-to-Golgi protein traffic. The complete disappearance of the acidic vesicles from the cells of the division and elongation zones as well as the arrest of cell elongation become visible later. Recent studies revealed that PCD, as the regulated mechanism of plant cell death, developed upon application of tenuazonic and cyclopaldic acids [80,81]. With regard to STA, our hypothesis requires careful examination.

Finally, STA is suggested to represent a potent mitochondrial toxin, consistent with its non-selective bioactivity. Such toxins should rather be eliminated from herbicide screening; however, some of them are potential antitumor agents [82,83].

An alternative mechanism can be deduced for STA from data previously reported for DHC (**9**), one of the most studied macrolides regarding its mechanism of action including phytotoxicity. DHC caused light-dependent electrolyte leakage from cucumber leaf discs [9], led to 15% loss of chlorophyll *a* in leaf segments of *Digitaria sanguinalis*, acted as a mitotic disruptor in garlic root tips and inhibited ATP synthesis and Mg^2+^-ATPase activity in *D. sanguinalis* thylakoids. The authors concluded that the action of DHC affects photosynthesis or interferes with the synthesis of part of the photosynthetic apparatus [8]. Recently the molecular target of DHC in Jurkat cells was discovered; ATP-citrate lyase (ACL) was demonstrated to be inhibited in vitro and in a cell-based model [79]. Notably, cell death as a consequence of ACL inhibition is mediated by accumulation of ROS [84]. ACL activity was detected in plants, fungi, animals and bacteria [85]; in plants it plays a role in the generation of cytosolic acetyl-CoA [86]. The effect of DHC on plant ACL was not studied, but it is likely that the impact of the toxin on this enzyme can explain its wide spectrum of biological activity. The toxicological and physiological profile of DHC is similar to those of STA demonstrated in our work (phytotoxicity, wide spectrum of biological activity, impact on pigment content, electrolyte leakage and mitosis).

As discussed above, the MOA of HBI (**2**) is likely different from that of STA (**1**). Application of HBI caused formation of green islands on wheat leaves similar to PNN [23,87]. As shown is this study, HBI treatment strongly decreased photosynthetic pigment content in *A. thaliana* leaves under the light condition, presumably inhibiting their biosynthesis (Figure 4B). However, in another study, HBI was shown to interact with bovine-brain calmodulin and to inhibit the activation of the calmodulin-dependent enzyme cAMP phosphodiesterase. Among the various known natural calmodulin inhibitors, HBI (**2**) was a potent compound, displaying noticeable phytotoxicity [12,13,88]. Both calmodulin and cAMP occur in plants and could represent molecular targets of HBI and other fungal toxins, such as ophiobolin A, which interacts with calmodulin of both animal and plant origin [89]. In plants, cAMP can act as an integrator of various signals with a role in the coordination of systemic responses, such as the reactions to biotic and abiotic stresses [90,91]. Pinolidoxin (**4**), a compound structurally similar to HBI (**2**), inhibits the activity of elicitor-induced phenylalanine-ammonia-lyase in poplar cell cultures, which can be correlated with low cAMP levels [92].

## 4. Conclusions

Our study of the effects of stagonolide A and herbarumin I on physiological and biochemical parameters of sensitive plants suggests that they have different mechanisms of action (MOA). These MOA resemble the toxic effects of well-known macrolides, such as brefeldin A, dehydrocurvularin and pyrenophorol.

Stagonolide A is proposed to inhibit the intracellular vesicular traffic from the endoplasmic reticulum to the Golgi. However, this hypothesis needs further support by cytological studies, protein quantification, etc. Molecular targets of STA can be elucidated using BFA as a positive control.

HBI presumably affects the biosynthesis of photosynthetic pigments and/or cAMP phosphodiesterase. These hypotheses could be further evaluated by quantification of isoprenoid and porphyrins, and by the respective enzyme inhibition assays.

Finally, HBI is likely a plant-specific toxin and therefore more prospective for further herbicide development than non-selective STA. However, if the latter turns out to target a novel molecular site, it will represent an interesting biochemical tool for studies on membrane trafficking similar to brefeldin A.

## 5. Materials and Methods

### 5.1. Material

#### 5.1.1. Fungal Phytotoxins and Herbicides

**Figure 12:**
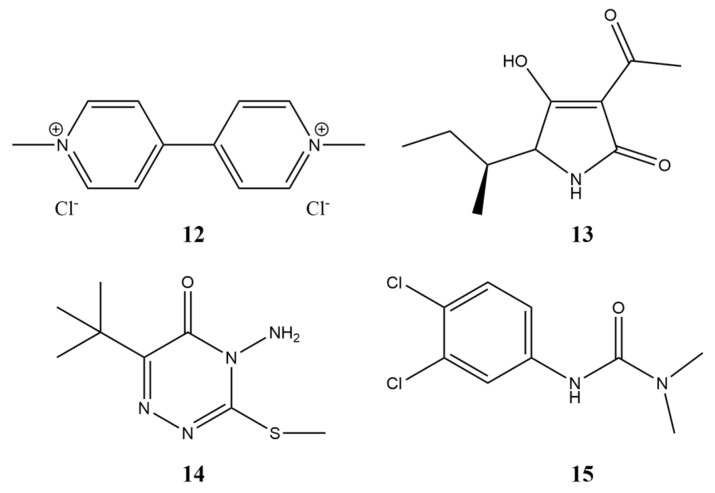
The structure of compounds used in this study for positive control. **12**—paraquat, **13**—tenuazonic acid, **14**—metribuzin, **15**—diuron.

The fermentation of the toxin-producing fungus *Stagonospora cirsii* S-47, as well as extraction and purification of stagonolide A (**1**, Figure 1) and herbarumin I (**2**, Figure 1), were performed as described [16]. Tenuazonic acid (**13**, Figure 12) was isolated from culture filtrate of *Alternaria tenuissima* according to [93]. Commercial herbicides with different modes of action used as positive controls in physiological tests were metribuzin, diuron, and paraquat (Sigma-Aldrich, St. Louis, MO, USA) (Figure 12).

#### 5.1.2. Plants

For most bioassays, Canada thistle (Cirsium arvense (L.) Scop.) and Thale cress (*Arabidopsis thaliana* (L.) Heynh., ecotype Col-0, Arabidopsis) plants were used. The first species is estimated as a primary control target while the latter is commonly used and recommended for mode of action studies [94].

Seeds and underground shoots of *C. arvense* were collected in the experimental field of the All-Russian Institute of Plant Protection (Saint-Petersburg, Russian Federation). Seeds of Arabidopsis were obtained from the Arabidopsis Biological Resources Centre (Ohio State University, Columbus, OH, USA). Spinach (*Spinacia oleracea* L.) plants in individual pots with a peat/sand mixture as well as bulbs of a commercial variety of common onion (*Allium cepa* L.) were obtained from a local market.

For leaf bioassays, cuttings (about 5 cm in length) of underground *C. arvense* shoots were planted in pots with a soil mixture (peat–sand, 3:1 *v*/*v*) and incubated in a greenhouse at approximately 24 °C for 3–4 weeks. Arabidopsis plants were grown from surface-sterilized seeds and were cultivated under the greenhouse conditions for 4 weeks.

For root bioassays, *C. arvense* seedlings were obtained from surface-sterilized seeds in Petri dishes with moistened filter paper. Arabidopsis seedlings were grown in the dishes with MS medium [95] supplemented with 1% sucrose and 0.35% phytagel (Sigma-Aldrich). The seedlings of both plants were incubated in a growth room under the continuous light of luminescent lamps with a photosynthetic photon flux density of 70–100 µmol/m^2^s at 24 °C. For the determination of mitotic disorders after toxin treatment, equally-sized healthy onion bulbs were germinated in 20 mL glass flasks containing tap water for 48 h at room temperature until the root length reached 1–2 cm.

### 5.2. Leaf Bioassays

#### 5.2.1. Leaf Puncture Assay

The toxin samples to be assayed were dissolved in 20 µL of DMSO and adjusted to a volume of 400 µL with distilled water. The final concentration of DMSO was 5% (*v*/*v*), the concentrations of the tested toxins were in the range of 0.25 to 2.00 mg/mL and 5% DMSO was used as control treatment. Punctured leaf discs of *C. arvense* and Arabidopsis were placed in two wet chambers and each disc was treated with 10 µL of test solution [23]. One chamber was kept in darkness, the other was kept at continuous light with a photosynthetic photon flux density of 70 µmol/m^2^·s.

Eight leaf discs taken from eight different plants (replicates) were used for each variant, and the experiment was performed twice. The results were photographed 48 h after treatment; the area of the necrotic lesions was measured using ImageJ 1.44 (Wayne Rasband, National Institutes of Health, Bethesda, MD, USA).

#### 5.2.2. Quantification of Photosynthetic Pigments

The toxin-treated (2 mg/mL) and control (untreated) leaf discs were collected after the phytotoxicity measurements (i.e., after 48 h of treatment). Each leaf disc was transferred to a centrifuge tube and frozen at −83 °C until analysis. To analyze the effect of STA and HBI on the contents of chlorophylls *a* and *b* and of carotenoids, the discs were extracted with 100% acetone as described by Lichtenthaler and Buschmann [96]. The absorbance of pigment extracts was measured with Beckman Coulter DU800 spectrophotometer (Beckman Coulter, Fullerton, CA, USA) at 470, 645, 662 and 750 nm. Pigment contents in each disc were calculated using the following formulae:

Chlorophyll *a* (c_a_, μg/mL) = 11.24 A_662_ − 2.04 A_645_

Chlorophyll *b* (c_b_, μg/mL) = 20.13 A_645_ − 4.19 A_662_

Carotenoids (c_x+c_, μg/mL) = (1000 A_470_ − 1.90 c_a_ − 63.14 c_b_)/214

Chlorophyll *a* (μg/cm^2^) = c_a_*V/S

Chlorophyll *b* (μg/cm^2^) = c_b_*V/S

Carotenoids (μg/cm^2^) = c_x+c_*V/S,

where V—volume of acetone (5 mL), and S—area of leaf disc (0.78 cm^2^) [97].

Five leaf discs (replicates) were used per treatment, and the experiment was re-peated twice.

#### 5.2.3. Electrolyte Leakage Assay

The effect of STA and HBI, respectively, on plasma membrane integrity was tested by measuring electrolyte leakage from toxin-treated punctured leaf discs [55,98]. The phytotoxicity assay was slightly modified to prevent inaccurate results due to electrolyte leakage into filter paper in case of severe destruction of leaf discs. The treatment was performed in small Petri dishes (40 mm in diameter) as wet chambers containing two layers of filter paper moistened with distilled water. Three leaf discs were placed in each Petri dish, damaged in the center with a pipette tip and treated with toxin solution with a concentration of 2 mg/mL. Plates were incubated under the same conditions as used for the phytotoxicity assay. After 48 h of treatment, the Petri dishes were filled with 5 mL of distilled water and incubated at room temperature for 1 h. Then, the bathing medium was transferred into small flasks and was used to measure the ion efflux using a conductivity meter, SevenEasy™ S30 (Mettler Toledo, Greifensee, Switzerland). Untreated discs boiled in bathing medium were used as positive control.

Three dishes containing three discs taken from three different plants (replicates) were used for each treatment, and the experiment was conducted twice.

#### 5.2.4. Chlorophyll Fluorescence Measurement

Light-induced transients of fast fluorescence (OJIP-kinetics) in leaves were measured using a Dual-PAM-100 (Walz, Effeltrich, Germany) and Dual PAM v1.19 software (Walz, Christof Klughammer) in the mode of single-beam excitation of polyphasic fluorescence during 300 ms. Solutions of STA or HBI in 1% DMSO at a final concentration of 2 mg/mL were applied to the surface of punctured leaf discs (d = 12 mm). The leaf discs were placed in transparent chambers on 3 layers of wet filter paper and wrapped up with polyethylene film to prevent the drying of leaf material. The chambers were placed in a growth room on a rack shelf under constant illumination with daylight fluorescent lamps with a PAR flux density of 90–100 µmol/m^2^s. An aqueous solution of an inhibitor of electron transport in PSII with a known mechanism of action [99], diuron, at a concentration of 200 µM, was used as positive control, while 1% DMSO was used as negative control. Ten leaf discs taken from ten different plants (replicates) were used for each variant. OJIP-kinetics were studied 6, 24 and 48 h after application of phytotoxins. Before measurements, leaf discs were dark-adapted for at least 30 min to achieve full re-oxidation of Q_A_^−^ and complete inactivation of ATP syntase, FNR, Rubisco and other enzymes of the Calvin–Benson cycle. OJIP measurements were performed using saturating red light of 3000 µmol m^−2^ s^−1^ intensity, with pulse width 80 µs, with 128,000 points per record and 300 ms polyphasic fluorescence trigger mode (Fast Acquisition Mode). Raw fluorescence transients (mV) were recorded with a data acquisition rate of 1 point per 10 µs. The intensity of measuring light was 24 µmol m^−2^ s^−1^ and a high modulation frequency of measuring light was applied. Recorded DUAL-PAM 100 data in CSV format were analysed using pyPhotoSyn software [100]. The fluorescence signal at 40 µs after the onset of illumination was considered as F_0_.

To depict the fluorescence transients, raw Chl *a* fluorescence rise kinetics were normalized using F_0_ and Fm as the relative variable fluorescence V(t) = (F(t) − F_0_)/(F_m_ − F_0_), where F_0_ means the minimal fluorescence when all PSII RCs are open; Fm means maximal fluorescence when all PS II RCs are closed; F(t) means fluorescence at time t after onset of actinic illumination [8]. OJIP-curves were plotted on a logarithmic time scale from 40 µs to 1 s. The characteristics points of photo-induced chlorophyll fluorescence transients were used to calculate specific characteristics of the light phase of photosynthesis according to the JIP-test algorithm developed by Strasser et al. [101,102], such as F_V_/F_m_ maximum quantum yield for primary photochemistry, F_V_/F_0_ maximum ratio of quantum yields of photochemical and concurrent non-photochemical processes in PSII, and performance index on absorption basis PI_ABS_.

The experiment was repeated thrice.

#### 5.2.5. Hill Reaction

The Hill reaction activity was measured spectrophotometrically as the rate of ferrocyanide formation according to Avron and Shavit [103], with some modifications. Thylakoids were isolated from spinach as described by Whitehouse and Moore [104]. The chlorophyll concentration was determined spectrophotometrically [105]. The reaction mixture included the “osmotically shocked” chloroplasts in an amount equivalent to 50 µg of chlorophyll, which were resuspended in the buffer containing 330 mM sorbitol, 50 mM HEPES (pH 7.6), 2 mM EDTA, 1 mM MgCl_2_, 1 mM MnCl_2_ and 0.5 mM K-ferricyanide (K_3_Fe(CN)_6_). STA or HBI was added to the experimental samples at one of the three concentrations examined (50, 100 and 200 µg/mL, respectively). Diuron (200 µM) was used as a positive control. The experimental samples were illuminated with a metal halide lamp (PAR 1000 µmol/m^2^s) for 15 min. The control samples without the addition of phytotoxins were exposed to the light for 15 min, while the “dark” control was left for 15 min in the dark. At the end of the incubation period, trichloroacetic acid was added to all variants to a final concentration of 2%. Inactivated chloroplasts were precipitated by a short centrifugation (12,000× *g*, 5 min), and the optical density of the supernatant was determined at 420 nm using a UV-2600 spectrophotometer (Shimadzu, Kyoto, Japan). The photochemical activity of chloroplasts (the rate of electron flow in ETC) was evaluated as µM ferrocyanide formation per hour per mg of Chl.

The experiments were carried out three times; in each experiment three analytical replicates of each composition of the reaction mixture were analyzed.

#### 5.2.6. ROS Assay

Semi-quantitative ROS analysis was conducted using fluorescent dyes specific either for superoxide (dihydroethidium) [106,107], or for singlet oxygen (Singlet Oxygen Sensor Green, SOSG) [108], or for peroxides (CM-H_2_DCFDA) [109]. All the dyes were from Thermo Fisher Scientific (Waltham, MA, USA). Treatment of leaf discs with STA and HBI was carried out as described above (5.2.3–5.2.5). A quantity of 1% DMSO was used as a negative control, while paraquat (10 µM) was used as a positive control when analyzing the production of superoxide and peroxides, and tenuazonic acid (2 mM) and metribuzin (250 µg/mL) were used as positive controls when analyzing the production of singlet oxygen. After application of the substances, the leaf discs of *C. arvense* and *A. thaliana* were incubated for 6 or 24 h in the dark or in the light (∼130 µmol/m^2^s). At the next step, the discs were infiltrated with a dye (2 µM dihydroethidium, 5 µM SOSG or 5 µM CM-H_2_DCFDA, respectively) using a syringe, and incubated for further 2 h under the same conditions as described by [110]. Then the discs were analyzed with an Olympus BX51 fluorescence microscope (Olympus Corporation, Japan) equipped with BP 460–495, DM 505, BA 510–550 filters. The images were obtained with a colorView II digital camera, the exposure time was 200 ms for SOSG and CM-H_2_DCFDA and 500 ms for dihydroetidium. After receiving the images, the average fluorescence intensity of the dyes was calculated using ImageJ software 1.50i (Wayne Rasband, National Institutes of Health, Bethesda, MD, USA).

In each treatment, two independent experiments were conducted with at least eight samples.

### 5.3. Root Bioassays

#### 5.3.1. Analysis of pH in Intracellular Structures and in the Apoplast Using Confocal Laser Scanning Microscopy

Acridine Orange (AO; Invitrogen, Waltham, MA, USA) was used as a vital dye that penetrates cells. It stains nucleic acids and simultaneously, having pH-sensing properties, visualizes acidic intracellular structures including autophagosomes. The experiments were carried out on 10–12-day old Arabidopsis seedlings. The experimental groups of seedlings were incubated in phytotoxin solutions containing 100 mM sorbitol and 0.1% DMSO, supplemented with either 10 µg/mL STA or 50 µg/mL HBI. These concentrations of phytotoxins are known as non-lethal but inhibiting root growth [24]. After incubation in toxin-free control or in toxin-supplemented experimental solutions, the seedlings were transferred to an aqueous solution of 25 µM AO for 10 min, then washed for 1 min in a buffer of 100 mM sorbitol, 2 mM Tris, 1 mM MES, 0.1 mM KCl, 0.1 mM CaCl_2_, pH 6.0 and analyzed under a confocal microscope LSM 780 (Carl Zeiss, Oberkochen, Germany) with a Plan-Apochromat objective 40×/1.4 Oil DIC. AO fluorescence was excited by a 488 nm argon laser; the fluorescence signal was detected in two channels, red and green (615–660 nm and 530–540 nm, respectively). To validate the method for assessing the pH of the apoplast using the AO fluorescent dye, experiments were performed in which the plasmalemma H^+^-ATPase inhibitor sodium orthovanadate (Na_3_VO_4_, Sigma-Aldrich) was used as a positive control.

#### 5.3.2. Mitochondrial Membrane Potential Assay

To determine the changes in Δ*Ψ*m, a system of two fluorescent membrane potential-sensitive dyes was used: DiBAC_4_(3) (bis-1,3-dibutylbarbituric acid trimethine oxonol) and TMRM (tetramethylrodamine methyl ester). The lipophilic anionic dye DiBAC_4_(3) was used as a potential sensor of the plasma membrane Δ*Ψ*_P_, and the lipophilic cationic dye TMRM served as a probe of the mitochondrial potential Δ*Ψ*m. As a pharmacological control of the sensitivity of the Δ*Ψ*m evaluation method, experiments were carried out where Arabidopsis seedlings were treated with the mitochondrial ATP synthase inhibitor oligomycin at 10 µM and the protonophore CCCP (carbonyl cyanide *m*-chlorophenyl hydrazone) at 25 µM. The roots of 7–10-day-old seedlings were incubated in solutions in the presence or in the absence of phytotoxins (as in Section 5.3.1), and simultaneously were stained by the adding of the membrane potential-sensitive dyes (250 nM DiBAC_4_(3) or 100 nM TMRM). After that seedlings were studied in incubation medium without any washing under a confocal laser scanning system LSM 780 (Carl Zeiss, Jena, Germany) with a Plan-Apochromat 40×/1.4 Oil DIC objective. Fluorescence was excited by a 488 nm argon laser; the signal was detected in the range of 535–605 nm for TMRM and 500–545 nm for DiBAC_4_(3), respectively. Automatic segmentation of microscopic images was performed using machine learning algorithms of the Zen Intellesis software module of the ZEN software v.2.3 (Carl Zeiss, Germany), and the average fluorescence intensities of the TMRM probe per µm^2^ mitochondria of control and experimental root cells were calculated.

#### 5.3.3. Mitotic Index

The mitotic index in the tips of onion rootlets was estimated using a common technique [29]. The tips of five roots 1 cm long were cut off and kept in STA solution (concentration 0.1–10 µg/mL) in 2% ethanol for 2 h. In the control, the roots were kept in 2% ethanol. The treated roots were fixed in Clark’s liquid (3 parts of 96% ethanol, 1 part of glacial acetic acid) for 5 min at room temperature and stored at −18 °C for 2–3 days. The nuclei in the samples were stained with acetocarmine for 2 min after heating and boiling, after which the samples were kept for 20 min in 45% acetic acid. Then, after removing the epidermis, the tip of the root was cut 1–2 mm long and crushed on a slide in a drop of 45% acetic acid by slightly pressing on the cover glass. The resulting monolayers of cells were observed under a light DM2500 microscope equipped with an N Plan 40×/0.65 Ph2 objective and a DFC320 digital camera (Leica Microsystems, Wetzlar, Germany). The number of dividing cells in different phases was counted in 30 fields of vision of 100 cells each. The mitotic index (MI) was determined by the formula MI = Nd/N*100%, where Nd is the number of dividing cells and N is the total number of registered cells.

#### 5.3.4. Tubulin and Actin Patterns

Roots of 5-day-old *C. arvense* seedlings treated for 24 h with STA at a concentration of 10 µg/mL were used for actin and tubulin immunolocalization. According to previously-described processes, 8 mm long root tips were fixed, sectioned and processed [111,112]. Longitudinal vibratome sections were incubated with mouse anti-tubulin IgG clone DM1A or mouse monoclonal anti-actin (*Zea mays*) antibody (Sigma-Aldrich); Alexa Fluor 488 goat anti-mouse IgG (Invitrogen) secondary antibodies were used. Sections were analyzed using a confocal microscope LSM 780.

### 5.4. Statistical Analysis

Statistical significance of the effects was estimated using one-way Analysis of Variance (ANOVA) and multiple comparisons with Tukey’s post-hoc test at the level of *p* < 0.05 using Statistica 12 (Dell, Round Rock, TX, USA). The statistical analysis was interpreted using the compact letter display methodology to clarify the presentation of results in figures; the same letters indicate statistically indistinguishable means. For some pairwise comparisons, Student’s *t*-test (*p* < 0.05) was used.

## Figures and Tables

**Table 1:** Changes in the relative levels of fluorescence signal (%) of ROS-sensitive dyes in *Cirsium arvense* and *Arabidopsis thaliana* leaf discs treated with phytotoxins compared to mock control.

Treatment	*Cirsium arvense*				*Arabidopsis thaliana*			
	Dark, 6 h	Light, 6 h	Dark, 24 h	Light, 24 h	Dark, 6 h	Light, 6 h	Dark, 24 h	Light, 24 h
Superoxide-specific fluorescence								
Stagonolide A	17	0	33 *	28 *	38 *	29	75 *	24
Herbarumin I	11	14	26 *	38 *	39 *	33	52 *	18
Paraquat	12	37 *	31 *	32 *	28 *	43 *	51 *	40 *
Peroxide-specific fluorescence								
Stagonolide A	132 *	64 *	27	58 *	63*	67	49 *	25
Herbarumin I	91	47 *	30	56 *	8	48	68 *	58 *
Paraquat	65	39	68 *	96 *	−11	112 *	43 *	82 *
Singlet oxygen-specific fluorescence								
Stagonolide A	4	34	−2	34	38 *	29	21	3
Herbarumin I	−4	13	−5	25	36 *	49	21	-3
Tenuazonic acid	36 *	46 *	20	55 *	40 *	58 *	13	15
Metribuzin	9	22	1	30	38 *	62 *	22	23

*—values significantly differ from mock control (*t*-test, *p* < 0.05).

**Table 2:** The effects of stagonolide A treatment at different concentrations on mitotic index in onion root tips.

Concentration, µg/mL	The Proportion of Dividing Cells at Different Phases (% of the Total Number of Cells)				Mitotic Index, %	Disorders
	Prophase	Metaphase	Anaphase	Telophase		
Control (mock)	7.37	1.75	0.88	1.23	11.4 a	No disorders
0.1	2.04	2.04	4.07	1.48	9.7 a	Binucleated cells
1	1.53	0.59	2.12	0.35	5.4 b	Binucleated cells
10	1.53	0.94	3.53	0.12	6.2 b	Nuclear fragmentation

Different letters indicate significant differences between treatments (*p* < 0.05, Tukey’s post-hoc test).

## Data Availability

The data are available upon the request.

## Supplementary Materials

The following supporting information can be downloaded at: https://www.mdpi.com/article/10.3390/toxins15040234/s1, Figure S1: The Hill reaction rates determined in spinach chloroplasts which were treated by diuron or different concentrations of stagonolide A and herbarumin I; Figure S2: Fluorescence of DHE determined in *C. arvense* and *A. thaliana* leaves; Figure S3: Fluorescence of CM-H_2_DCFDA determined in *C. arvense* and *A. thaliana* leaves; Figure S4: Fluorescence of SOSG determined in *C. arvense* and *A. thaliana* leaves; Figure S5: The changes in the apoplastic pH of Arabidopsis root cells after treatment with 1 mM sodium orthovanadate, an inhibitor of plasma membrane H^+^-ATPase; Figure S6: Patterns of actin and tubulin in the root apical meristem of *Cirsium arvense* seedlings: control and after 24 h treatment with 10 µg/mL stagonolide A; Table S1: Formulae and glossary of terms which were used for the analysis of OJIP transients in this research [40,101,102,113]; Table S2: Mean of fluorescence of *Cirsium arvense* and *Arabidopsis thaliana* leaf disks treated with phytotoxins followed by ROS-sensitive dyes incubated in the dark and under continuous light.
