# Supplementary material for: Effects of Phytotoxic Nonenolides, Stagonolide A and Herbarumin I, on Physiological and Biochemical Processes in Leaves and Roots of Sensitive Plants
Source: Toxins (Basel). 2023 Mar 23;15(4):234. doi: 10.3390/toxins15040234 (PMC10145764; doi:10.3390/toxins15040234)
Supplement: Supplementary file 1 [file toxins-15-00234-s001.zip › toxins-2222774-supplementary.pdf]

toxins

Supplementary Materials

Effects of Phytotoxic Nonenolides, Stagonolide A and Herbarumin I, on Physiological and Biochemical Processes in Leaves and Roots of Sensitive Plants

The following Supplementary Materials are available for this article:

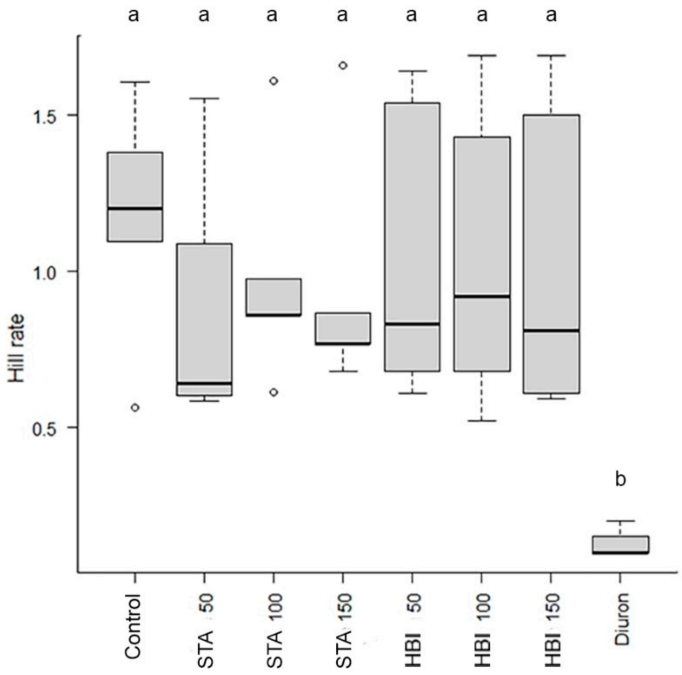

Figure S1. The Hill reaction rates determined in spinach chloroplasts which were treated by diuron or different concentrations of stagonolide A (STA) and herbarumin I (HBI). The box-plots show the minimum value, the first quartile, the median, the third quartile and the maximum value of 9 measurements per treatment from three independent experiments (n = 9). Outliers are shown as dots. Different letters indicate significant differences according to one-way ANOVA and multiple comparisons with Tukey's post-hoc test at the level of p < 0.05.

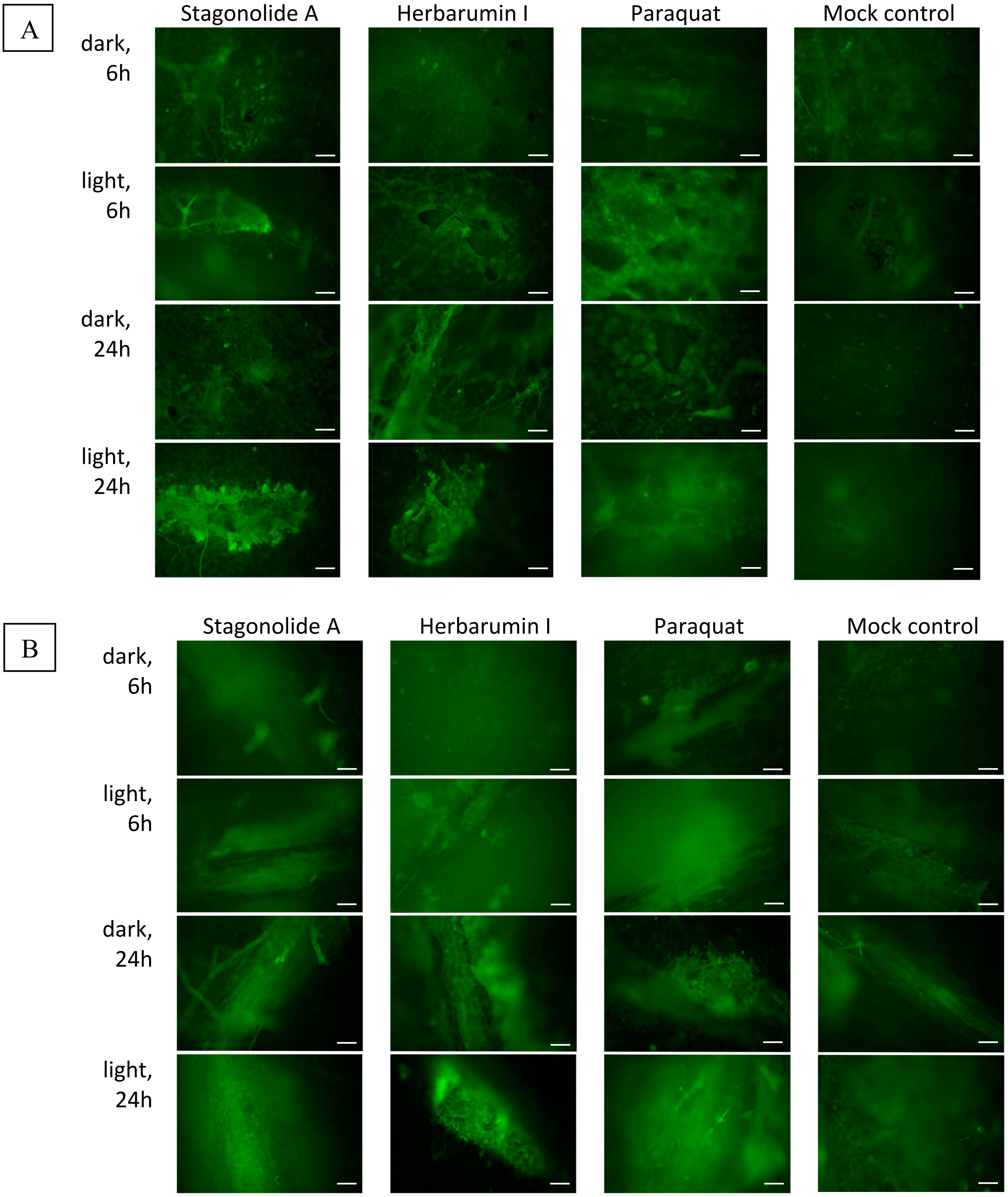

Figure S2. Fluorescence of specific for superoxide fluorescent dye dihydroethidium determined in *Cirsium arvense* (A) and *Arabidopsis thaliana* (B) leaves. Scale bar: 200 µm.

toxins

MDPI

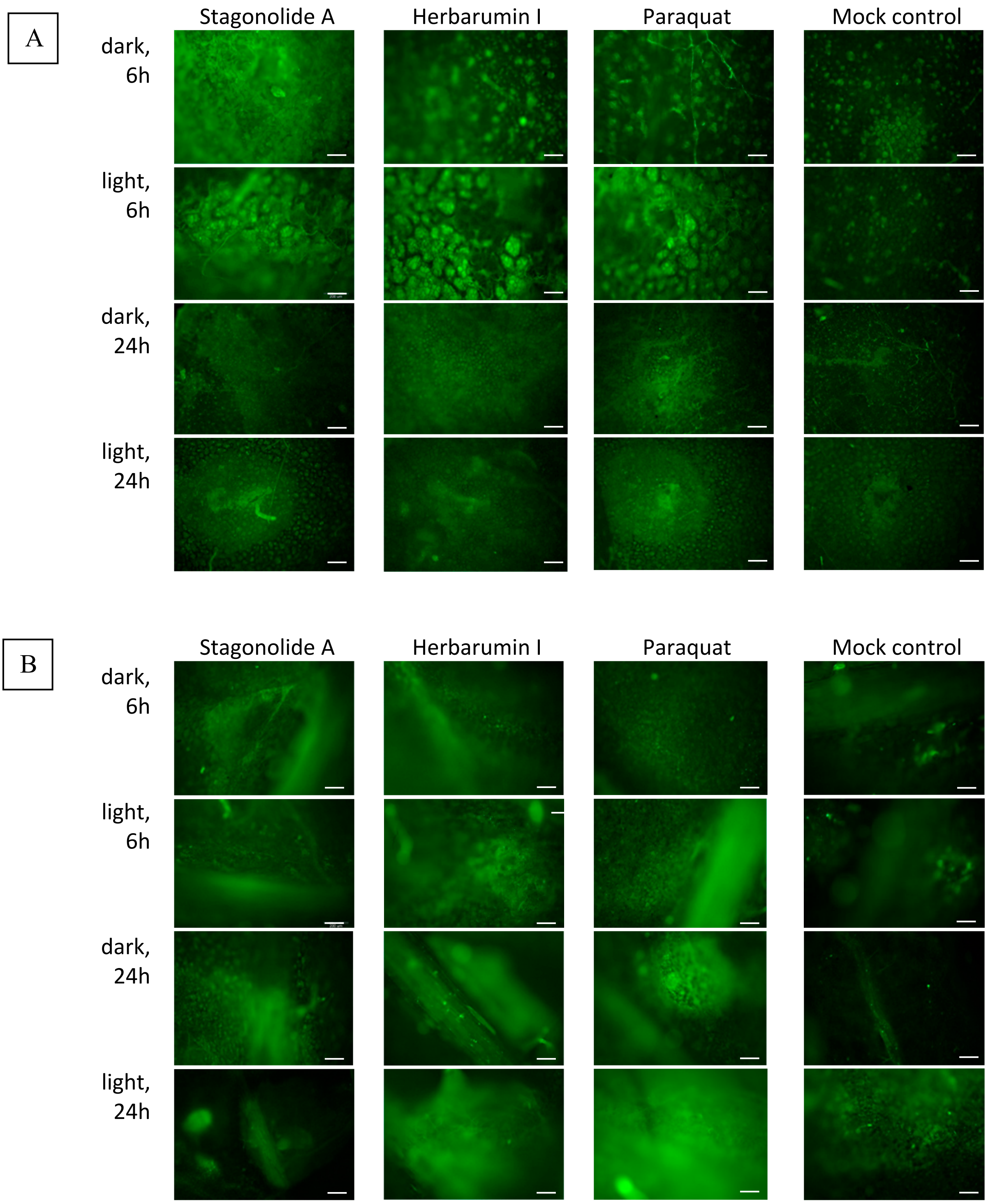

Figure S3. Fluorescence of specific for peroxides fluorescent dye CM-H_2DCFDA determined in *Cirsium arvense* (A) and *Arabidopsis thaliana* (B) leaves. Scale bar: 200 μm.

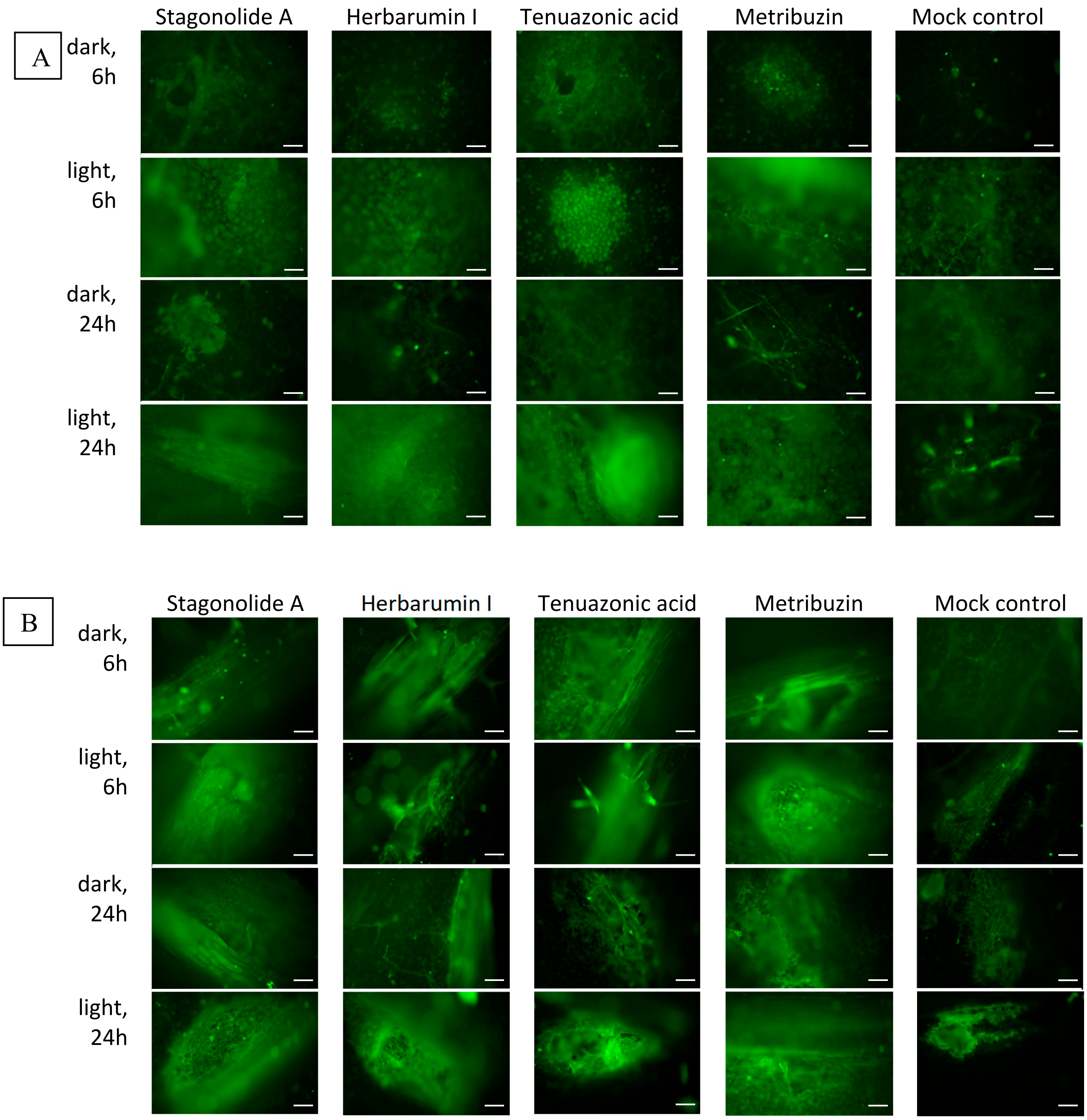

Figure S4. Fluorescence of specific for singlet oxygen fluorescent dye SOSG determined in *Cirsium arvense* (A) and *Arabidopsis thaliana* (B) leaves. Scale bar: 200 μm.

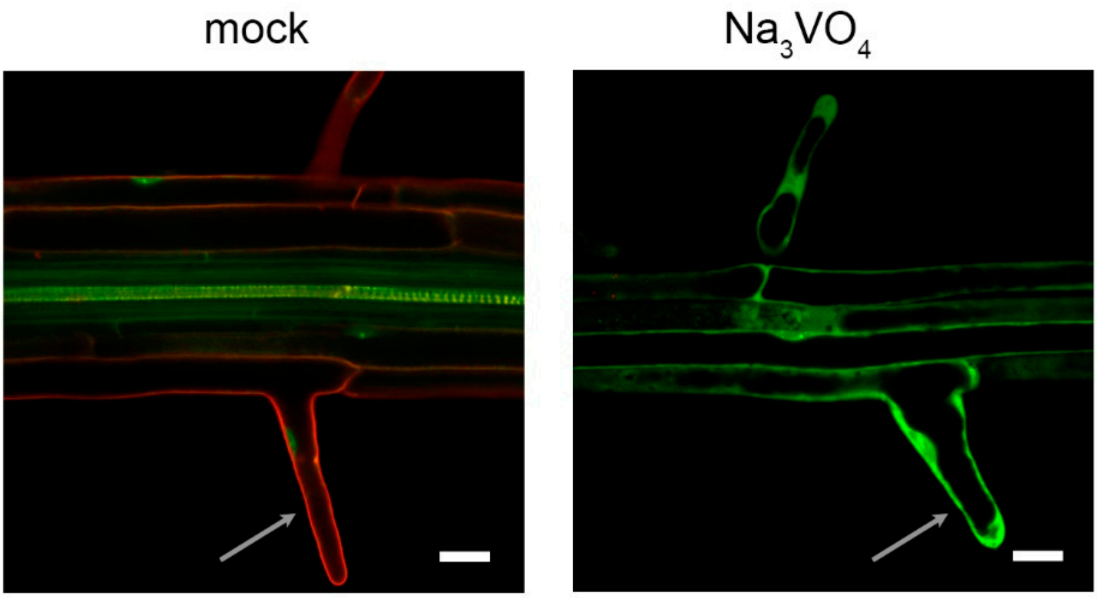

Figure S5. The changes in the apoplastic pH of Arabidopsis root cells after treatment with 1 mM sodium orthovanadate, an inhibitor of plasma membrane H+-ATPase. Arabidopsis seedlings were treated for 15 min with 1 mM sodium orthovanadate in 100 mM sorbitol, 0.1% DMSO, pH 6.5 (Na_3VO_4 treatment) or incubated in the same solution in the absence of inhibitor (mock). Confocal laser scanning microscopy of single optical longitudinal section of Arabidopsis roots was performed. Merged images of red and green channels are shown. Red channel: λex: 488 nm, λem = 615 – 660 nm; green channel: λex: 488 nm, λem = 530 – 540 nm. Gray arrows point to the root hair plasma membrane and apoplast stained with acridine orange. Scale bars: 20 μm.

toxins

MDPI

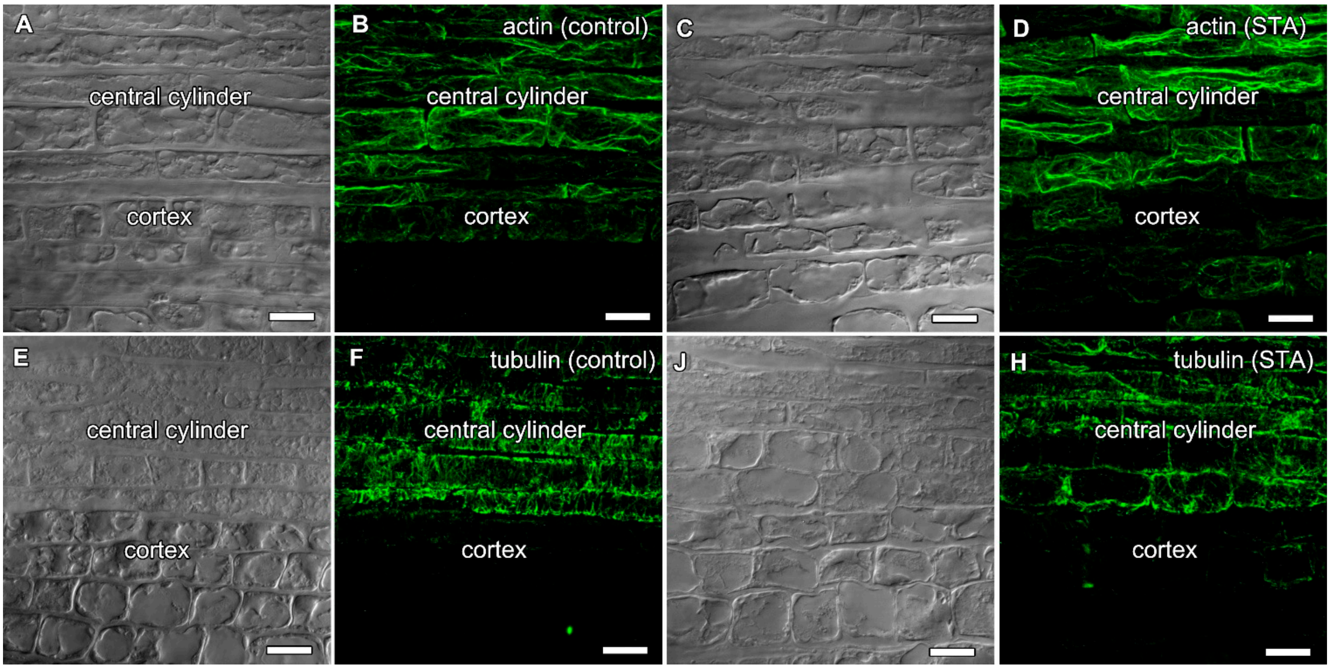

Figure S6. Patterns of actin (**A–D**) and tubulin (E–H) in the root apical meristem of *Cirsium arvense* seedlings: control (**A,B,E,F**) and after 24 h treatment with 10 µM stagonolide A (STA) (**C,D,J,H**). Immunolocalization of the actin and tubulin cytoskeleton (green channel), confocal laser scanning microscopy on longitudinal vibratome sections at a distance of 150 µm from the initial cells. Single optical sections with differential interference contrast (**A,C,E,J**) and maximum intensity projection of z-series of 40 optical sections, 8 µm in depth (**B,D,F,H**). Scale bars: 10 µm.

Table S1. Formulae and glossary of terms which were used for the analysis of OJIP transients in this research (Strasser et al. 2004; Strasser et al. 2010; Goltsev et al., 2016; Stirbet et al., 2018)

Technical fluorescence parameters	
F_t	Fluorescence at time t after onset of actinic illumination
F_0	Minimal fluorescence (all PSII RCs are assumed to be open)
$F_P = F_m$	Maximal recorded fluorescence intensity, at the peak P of OJIP
$F_V = F_t - F_0$	Variable fluorescence at time t
$F_V = F_m - F_0$	Maximal variable fluorescence
$V_t = (F_t - F_0)/(F_m - F_0)$	Relative variable fluorescence at time t
F_V/F_0	The ratio between the rate constants of photochemical and non-photochemical deactivation in PSII
$V_J = (F_J - F_0)/(F_m - F_0)$	Relative variable fluorescence at the J-step
$M_0 = 4(F_{300\mu s} - F_0)/(F_m - F_0)$	Approximated initial slope (in ms^{-1}) of the fluorescence transient normalized on the maximal variable fluorescence F_V
Energy fluxes	
ABS	The photon flux absorbed by the antenna of PSII units
TR	The part of ABS trapped by the active PSII units that leads to Q_A reduction
DI	The part of ABS dissipated in PSII antenna in processes other than trapping
ET	The energy flux associated with the electron transport from Q_A^- to the intersystem electron acceptors
RE	The energy flux associated with the electron transport from Q_A^- to the final electron acceptors of PSI
Specific energy fluxes (per Q_A^--reducing PSII reaction center - RC)	
$ABS/RC = M_0\ (1/V_J)(1/\varphi_{Po})$	Absorption flux (of antenna Chls) per RC (also a measure of PSII apparent antenna size)
$TR_0/RC = M_0\ (1/V_J)$	Trapped energy flux (leading to Q_A reduction) per RC
$ET_0/RC = M_0\ (1/V_J)(1 - V_J)$	Electron transport flux (further than Q_A^-) per RC
$RE_0/RC = M_0\ (1/V_J)(1 - V_I)$	Electron flux reducing end electron acceptors at the PSI acceptor side, per RC
$DI_0/RC = ABS/RC - TR_0/RC$	The flux of energy dissipated in processes other than trapping per active PSII
Efficiencies and quantum yields	
$\psi_{E0} = ET_0/TR_0 = 1 - V_J$	Efficiency with which a PSII trapped electron is transferred from Q_A^- to PQ
$\psi_{R0} = RE_0/TR_0 = 1 - V_I$	Efficiency with which a PSII trapped electron is transferred to final PSI acceptors
$\varphi_{Po} = Fv/Fm = TR_0/ABS = [1 - (F_0/F_M)]$	Maximum quantum yield for primary photochemistry
$\varphi_{E0} = ET_0/ABS$	Quantum yield of electron transport from Q_A to PQ
Performance indexe (products of terms expressing partial potentials at steps of energy bifurcations)	
$PI_{ABS} = (RC/ABS) \times [\varphi_{Po}/(1 - \varphi_{Po})] \times [\psi_{E0}/(1 - \psi_{E0})]$	Performance index (potential) for energy conservation from exciton to the reduction of intersystem electron acceptors

References:

1. Strasser, R.J.; Tsimilli-Michael, M.; Srivastava, A. Analysis of the Chlorophyll a Fluorescence Transient. In *Chlorophyll a Fluorescence*; Advances in Photosynthesis and Respiration 2004; Papageorgiou Govindjee, G.C., ed.; Springer: Dordrecht, The Netherlands, 2004; volume 19. https://doi.org/10.1007/978-1-4020-3218-9_12.
2. Strasser, R.J.; Tsimilli-Michael, M.; Qiang, S.; Goltsev, V. Simultaneous in vivo recording of prompt and delayed fluorescence and 820-nm reflection changes during drying and after rehydration of the resurrection plant *Haberlea rhodopensis*. *Biochim. Biophys. Acta (BBA) Bioenerg.* **2010**, *1797*, 1313–1326. https://doi.org/10.1016/j.bbabio.2010.03.008
3. Goltsev, V.N.; Kalaji, H.M.; Paunov, M.; Bąba, W.; Horaczek, T.; Mojski, J.; Kociel, H.; Allakhverdiev, S.I. Variable chlorophyll fluorescence and its use for assessing physiological condition of plant photosynthetic apparatus. *Russ. J. Plant Physiol.* **2016**, *63*, 869–893. https://doi.org/10.1134/S1021443716050058.
4. Stirbet, A.; Lazár, D.; Kromdijk, J., Govindjee Chlorophyll a fluorescence induction: Can just a one-second measurement be used to quantify abiotic stress responses? Photosynthetica 2018, 56, 86–104. https://doi.org/10.1007/s11099-018-0770-3

Table S2. Mean of fluorescence of Cirsium arvense and Arabidopsis thaliana leaf disks treated with phytotoxins followed by ROS-sensitive dyes incubated in the dark and under continuous light.

Treatment	*Cirsium arvense*				*Arabidopsis thaliana*			
	**dark, 6h**	**light, 6h**	**dark, 24h**	**light, 24h**	**dark, 6h**	**light, 6h**	**dark, 24h**	**light, 24h**
DHE								
Stagonolide A	25.3±2.8	23.1±2	25.2±3	29.5±5.4	25±2.6	27.1±3	31±5.3	29.3±4.5
Herbarumin I	24±2.5	26.4±4.7	23.9±1.4	31.7±3.8	25.2±4.7	28±5.4	27±1.9	27.9±3.2
paraquat	24.7±3.4	31.7±5.2	25±2.5	31.2±2.7	23.4±2.8	30±7.4	26.8±2.2	30.5±3.7
– control	21.6±4.7	23.1±7.4	19±3.5	23.6±3.6	18.3±2.6	21±1.3	17.7±1.5	23.6±2.7
CM-H_2DCFDA								
Stagonolide A	37.6±11.4	32.6±10	18±4.5	19.6±3.1	39.7±16	27.6±6.3	34.5±6.9	29.5±4.2
Herbarumin I	31±5.5	29.3±11.5	18.5±7.8	19.3±5.7	26.3±5.2	24.4±6.6	38.7±6.4	37.2±7.2
paraquat	26.7±11.6	27.7±7.3	23.9±4.4	24.3±2.8	21.7±5.7	35±14.5	33.1±4.2	43±15.1
– control	16.2±7.7	19.9±6.6	14.2±5	12.4±4.1	24.3±6.7	16.5±3.6	23.1±3.5	23.6±3.5
SOSG								
Stagonolide A	13.9±2.4	22±6	14.2±3.2	26.7±7.8	16.5±2	17.4±5.6	17.4±5.7	21.5±5.4
Herbarumin I	12.9±3.1	18.5±4.2	14±2.5	25±6.6	16.3±2.9	20.1±5	17.4±6	20.3±5.9
Tenuazonic acid	18.2±5.2	24.8±3.4	17.4±3.8	31±4.6	16.8±3.4	21.3±3.4	16.3±3.7	24±4.9
Metribuzin	14.6±3.5	20±5	14.7±1.6	26±4.8	16.5±3	21.9±7.2	17.5±4.8	25.6±6.1
– control	13.4±2.2	16.4±3.3	14.5±3	20±4.1	12±2.2	13.5±3	14.4±2.8	20.8±3.9
